# Integrating the secretome and interactome to identify novel biomarkers and therapeutic targets in colorectal cancer

**DOI:** 10.1186/s12964-025-02424-4

**Published:** 2025-10-10

**Authors:** Gabriel Henrique Caxali, Mirian Carolini Esgoti Aal, Catherine Wesselka Garcia Osvaldo, Jakeline Santos Oliveira, Lucas Tadeu Bidinotto, Robson Francisco Carvalho, Flávia Karina Delella

**Affiliations:** 1 Department of Structural and Functional Biology, Institute of Biosciences, São Paulo State University (UNESP), 250 Professor Doutor Antonio Celso Wagner Zanin St, Botucatu, SP 18618-689 Brazil; 2 Barretos School of Health Sciences Dr. Paulo Prata - FACISB, Barretos, SP Brazil; 3 Molecular Oncology Research Center, Barretos Cancer Hospital, Barretos, SP Brazil

**Keywords:** Colorectal cancer, Tumor microenvironment, Biomarkers, Molecular subtypes

## Abstract

**Background:**

Cancer is extensively studied, yet its origins and progression remain unclear. A key question is why tumors of the same type vary in aggressiveness and treatment response. In colorectal cancer (CRC), the third most common cancer, this variability led to the identification of molecular subtypes (CMS). However, the tumor microenvironment remains poorly understood and may be crucial for understanding carcinogenesis and drug resistance. A promising approach is analyzing cell interactions through ligand-receptor expression. This study used bioinformatics to examine CRC in different anatomical locations, identify diagnostic and prognostic biomarkers, and propose targeted drugs.

**Methods:**

Expression data were obtained from the TCGA-COAD database. All samples were filtered based on the tumor’s region of origin and purity. RNA-seq expression analysis was then conducted to assess molecular differences according to tumor location and purity, identifying region-specific ligands and receptors using the secretome list as a reference. Once these differences were identified, an interactome was constructed to depict cell interactions within the tumor microenvironment. The most relevant genes were then evaluated for their prognostic potential through survival analysis, and their susceptibility to pharmacological modulation was assessed to identify potential new drug candidates for CRC treatment.

**Results:**

The integration of secretome data and the construction of the interactome proved to be a valuable approach for detecting novel biomarkers specific to right- and left-sided CRC. Through this approach, *FGFR4*, *FLT1*, and *WNT5A* were identified as key biomarkers involved in tumor carcinogenesis, modulating distinct processes in each region, such as fibroblast recruitment and cell division. Based on these biomarkers, Dovitinib and Nintedanib were predicted as potential therapeutic agents, as they target multiple identified markers.

**Conclusion:**

This study highlights *FGFR4*, *FLT1*, and *WNT5A* as key diagnostic and therapeutic biomarkers for CRC, with their relevance varying based on the tumor’s site of origin. Leveraging these findings, we propose Dovitinib and Nintedanib as promising targeted therapies for CRC. These insights can enhance current treatment strategies and pave the way for future in vivo and in vitro studies, driving progress in CRC research and therapy.

**Supplementary Information:**

The online version contains supplementary material available at 10.1186/s12964-025-02424-4.

## Introduction

Cancer has been a major focus of research in recent years due to its complexity and high mortality rate, making it the second leading cause of death in the United States. Among the various types of cancer, colorectal cancer (CRC) stands out as a significant concern. CRC is the third most common cancer affecting both men and women in the United States, with an estimated 154,270 new cases and 52,900 deaths projected for 2025 [1].

The rising incidence of CRC is largely attributed to modern lifestyle and dietary changes, particularly decreased physical activity and the widespread adoption of the Western diet, which is high in fats and low in essential nutrients [2]. In addition to lifestyle factors, several biological and genetic aspects influence CRC risk, including age, race, gender, and genetic mutations [3]. Studies show that individuals over 75 face a higher risk; however, cases are increasingly being diagnosed in younger populations. Racial disparities also play a significant role, with African Americans exhibiting a greater predisposition—likely due to higher exposure to risk factors such as smoking, obesity, and physical inactivity. Gender differences further contribute to CRC incidence, with research consistently indicating a higher prevalence in men compared to women across various populations [4–6].

Certain molecular pathways are particularly significant in CRC due to their high incidence and critical role in carcinogenesis. These include the activation of the WNT signaling pathway and other pathways related to epidermal growth factor receptor (EGFR) signaling, such as PI3K/AKT, KRAS, and TGF-β pathways [7]. Recent studies continue to identify new genetic markers linked to CRC, including the mapping of short and long non-coding RNAs, DNA methylation of specific genes, and the discovery of novel genes associated with key processes, such as IGFBP-2, DKK3, and PKM2 [8].

While multiple molecular pathways contribute to CRC development, the disease typically follows a well-established sequence called the adenoma-carcinoma pathway [9, 10]. This process begins with abnormal cell proliferation in the intestinal mucosa, particularly within the crypts, leading to the formation of polyps. Over time, these polyps accumulate genetic mutations, acquire Malignant properties, and progress into adenomas or sessile serrated polyps. Adenomas account for approximately 70% of cases, while sessile serrated polyps Make up around 20%. A crucial Marker for detecting this progression is the APC gene, as mutations in this gene are present in roughly 70% of adenoma cases. Additionally, the microsatellite instability (MSI) pathway is Linked to Lynch syndrome, though it is less common, occurring in approximately 2% of cases [7, 8, 10–12].

Despite sharing common progression pathways, CRC remains a subject of intense research due to its distinct behavior in progression, survival, and drug resistance. Several studies have aimed to elucidate the behavioral differences of colorectal tumors based on their site of origin. It has been observed that pre-diagnostic symptoms such as anemia and abdominal pain are more commonly associated with tumors originating in the colon. In contrast, tumors present at diagnosis are often linked to alterations in bowel habits, including the presence of blood in the stool and changes in stool consistency [13]. This anatomical distinction also influences treatment strategies, as left-sided metastatic tumors have shown a better response to anti-EGFR monoclonal antibody therapy compared to right-sided tumors [14].

Large-scale studies have led to a consensus on the classification of CRC into four consensus molecular subtypes (CMS), each exhibiting unique behaviors driven by different signaling pathways [15, 16]. This classification is essential for improving prognostic, diagnostic, and therapeutic strategies. Advances in DNA and RNA sequencing have played a pivotal role in these discoveries, significantly enhancing our understanding of tumor biology. These breakthroughs have helped identify resistance mechanisms, develop neoantigens, and map genomic modifications associated with CRC [16, 17].

Following the establishment of the CMS classification, efforts were made to correlate site-specific factors with the molecular behavior of CRC [18]. This led to the identification of distinct molecular profiles associated with tumor location. Left-sided CRCs are predominantly classified as CMS2 and CMS4, which are generally associated with better prognoses and elevated expression of EGFR. In contrast, right-sided tumors exhibit higher frequencies of CMS1 and CMS3, which are linked to poorer prognoses, frequent BRAF mutations, MSI, and a high degree of CpG island methylator phenotype [19].

A major advancement in sequencing technology has been the improved understanding of tumor microenvironment (TME) dynamics. By identifying various cell types and subpopulations within tumors, researchers have gained deeper insights into the roles of inflammation and surrounding cells in cancer progression [20, 21]. Single-Cell RNA sequencing (scRNA-seq) has further refined this understanding by enabling a detailed analysis of cellular behaviors and interactions [22, 23]. One key approach in studying cell communication is interactome analysis, which examines ligand-receptor interactions to map complex cellular networks [24, 25]. In CRC, this analysis has helped elucidate the mechanisms behind different CMS, assess the roles of various cell types within the TME, and guide personalized drug development [26]. Ultimately, integrating interactome analysis in CRC research may lead to the discovery of novel biomarkers, identification of tumor site-specific characteristics, and advancements in personalized therapeutic interventions [27, 28]. Therefore, this study aimed to integrate secretome and interactome data with bulk RNA-seq analysis to gain deeper insights into CRC carcinogenesis, ultimately identifying potential new therapeutic targets and treatment strategies for this tumor.

## Materials and methods

### Choosing and downloading the reference secretome

The reference secretome List was obtained from version 23 of *The Human Protein Atlas* database (https://www.proteinatlas.org/) [29]. This List comprises 2,793 proteins found in extracellular compartments and the extracellular matrix, including those present in the blood, brain, digestive system, male and female reproductive systems, as well as immunoglobulins and membrane-associated proteins or proteins with unknown locations [30].

### Downloading and filtering expression data from bulk RNA-seq samples

We utilized expression data from The Cancer Genome Atlas (TCGA) Colon Adenocarcinoma (COAD) database (https://portal.gdc.cancer.gov/) [31], selected for its comprehensive clinical information on sample origins. Data acquisition and download were performed using the TCGAbiolinks v. 2.32.0 package [32], extracting raw counts from both normal and tumor samples. After downloading, the expression Matrices were filtered in R v. 4.4.1 based on the anatomical origin of the samples. Two distinct expression matrices were generated: one for samples from the right side of the large intestine (ascending and transverse colon) and another for the left side (descending colon, sigmoid, and rectum). To ensure data consistency, recurrent tumors and metastatic samples were excluded, retaining only normal tissue and primary tumor samples for analysis.

### High-Purity tumor sample filtering

After categorizing the samples based on tumor origin (right vs. left), we further filtered the expression matrices according to tumor purity. To accomplish this, we utilized the study by Aran, Sirota, and Butte [33], which assessed the purity of TCGA samples using four different methods (ESTIMATE, ABSOLUTE, LUMP, and IHC) and developed the Consensus Measurement of Purity Estimates (CPE). This approach allows for the identification of samples with a high proportion of tumor cells and minimal inflammatory infiltrate.

Samples classified as high-purity tumors were those with a CPE > 0.8. Following this filtering process, we generated four distinct expression matrices, categorized by tumor site (right vs. left) and tumor purity (high vs. low). The number of samples in each group is detailed in Table 1.

**Table 1:** Number of samples analyzed in each group in this study

Group	Number of Samples
Normal Tissue	41
Right High Purity	77
Right Low Purity	141
Left High Purity	71
Left Low Purity	74

### Differential expression analysis

To identify differentially expressed genes (DEGs) among the defined groups, we utilized the DESeq2 v.1.44.0 package [34]. Genes were considered significant if they met the criteria of Log2 Fold Change > |1.5| and adjusted *p*-value < 0.05. Following DEG identification, gene names were converted from ENSEMBL to Gene Symbols using the org.Hs.eg.db v.3.8.2 package [35]. All analyses were conducted in R v.4.4.

### Classification of samples by CMS

After stratifying the samples according to tumor location and purity, classification into the respective CMS was performed using the CMScaller package [36]. This tool assigns CMS subtypes based on the gene expression signatures defined by Guinney et al. [15], employing the Nearest Template Prediction (NTP) algorithm for subtype prediction [37].

### Identification of ligands and receptors and inference of cell communication

We identified ligands and receptors using the list described by Ramilowski [38], which includes 2,422 predicted ligand-receptor pairs in humans. To analyze cell communication, we applied the method proposed by Armingol [39] to identify upregulated genes functioning as ligands and receptors involved in the studied cellular process. Ligand-receptor pairs were considered significant if at least one component had a Log₂FC > 1.5 and was exclusive to a specific condition, allowing for the identification of condition-specific ligands. Finally, a comprehensive list was generated, containing the significant pairs along with their corresponding Log₂FC values and adjusted p-values.

### Protein-Protein interaction (PPI) networks of the identified pairs

Significantly altered ligand-receptor pairs were analyzed using the *Search Tool for the Retrieval of Interacting Genes/Proteins* (STRING) database (https://string-db.org) [40]. The analysis followed the parameters defined by Oliveira et al. [41], including the following settings: Network edges: Confidence; Active interaction sources: Experiments, Database, Co-expression, Neighborhood, and Co-occurrence; Minimum required interaction score: High confidence (0.700); Display simplifications: Hide disconnected nodes in the network. The resulting network was imported into *Cytoscape* 3.9.1 [42] using the *StringApp* extension. Finally, the network was clustered using the *CytoHubba* extension [43], where the ten most interconnected genes were identified using the degree algorithm.

### Functional enrichment analysis

The set of genes identified in the ligand-receptor pairs was analyzed for functional enrichment using the *EnrichR* platform (https://maayanlab.cloud/Enrichr/) [44]. Within this platform, we selected the *GO: Biological Process* database to examine gene ontologies and the *KEGG 2021* database to identify associated pathways. Only terms common to both members of the enriched pairs were considered. These selected terms were then highlighted in the *Protein-Protein Interaction (PPI)* network generated using the *STRING* tool.

### Survival analysis

Kaplan-Meier survival analysis was performed using the *Kaplan-Meier Plotter* tool (https://kmplot.com/analysis) [45]. We selected the colon cancer dataset, which includes microarray data from 1,336 patients. To assess survival differences based on tumor location, patients were categorized into two groups: right colon (237 patients) and left colon (233 patients). Additionally, we analyzed RNA-seq data from the pan-cancer dataset available on the same platform. For this analysis, we focused specifically on rectal adenocarcinoma patients (165 patients). Overall survival was used as the primary parameter in both analyses, with the median survival calculated for each group.

### Construction of ROC-Curves for target genes

To evaluate the sensitivity and specificity of the top 10 candidate genes in distinguishing between normal and tumor samples, receiver operating characteristic (ROC) curves were generated using the ROC Plotter platform (https://rocplot.org) [46]. The analysis was performed using a CRC dataset comprising 280 colon samples (101 non-responders and 179 responders) and 284 rectal samples (131 non-responders and 153 responders). ROC curves were constructed separately for each tumor site of origin.

### Validation of identified target genes by tumor site

To validate the target genes identified through differential expression and network analysis, the TNMplot tool (https://tnmplot.com) [47] was employed. This platform integrates RNA-seq and microarray data from 56,938 unique samples derived from publicly available datasets, including GEO, GTEx, TCGA, and TARGET. The “TNM-plot: Compare Tumor and Normal” function was utilized, and paired tumor–normal samples were selected to minimize bias. For RNA-seq analysis, colon adenocarcinoma and rectal adenocarcinoma samples were included. For microarray data, only colon samples were available.

### Analysis of sc-RNA-Seq data

Single-cell RNA sequencing (scRNA-Seq) data from Lee et al. [48] was used to identify cell types associated with the interactions predicted in the bulk RNA-Seq analysis. This dataset comprises 15 colorectal carcinoma samples collected from patients at the Samsung Medical Center in Seoul. The data were obtained from the *Curated Cell Cancer Atlas* (3CA) (https://www.weizmann.ac.il/sites/3CA/) [49]. Count data were processed using the *Seurat* package (v.5.1.0) in *RStudio* [50]. Differential expression analysis was conducted within the package to distinguish cell populations, followed by Principal Component Analysis (PCA). Principal components (PCs) 1 through 10 were used for graphical clustering at a resolution of 0.5, and Uniform Manifold Approximation and Projection (UMAP) was applied for cluster visualization. Distinct cell types were identified based on differential expression patterns, with various cell markers detected. To classify cell types using canonical markers (Table 2), we utilized the *CellMarker 2.0* platform (http://117.50.127.228/CellMarker/) [51]. The *dotplot* and *vlnplot* functions in *Seurat* (v.5.0.1) were used to evaluate expression profiles across different cell types.

### Verification of therapeutic targets using CRC genetic signatures

To identify potential therapeutic targets in *COAD/READ* datasets, we used *OCTAD* (http://octad.org/) [52], categorizing *TCGA* samples by tumor side and purity, with *GTEx* as controls. Genetic signatures were generated via *EdgeR*, selecting genes with *FC > 1.2* and *Adjusted p-value < 0.001*. The OCTAD platform then compared these signatures against its database (*19*,*127 tissue samples*,* 50 tumor types*,* 12*,*442 compounds*) to identify candidate drugs. Genes with the highest interaction scores in the *interactome* (via *cytoHubba*) were further analyzed using *DGIdb* (https://www.dgidb.org) [53], which compiles *30 databases* (*41*,*100 genes*,* 9*,*495 drugs*,* 29*,*783 interactions*). The *L1000FWD* platform (https://maayanlab.cloud/l1000fwd/) [54] was used to identify drugs reversing the expression profile (similarity score < 1). Finally, *ShinyDeepMap* [55] assessed the *10 most significant genes* from *PPI analysis*, measuring efficacy and selectivity using *CRISPR* and *shRNA* data from the *Cancer Dependency Map* [56].

### Data representation and analysis

Differentially expressed gene sets, including both unique and shared genes across groups, were identified using *Venny 2.1.0* (https://bioinfogp.cnb.csic.es/tools/venny/)*.* Volcano plots were generated with *VolcanoseR* (https://huygens.science.uva.nl/VolcaNoseR/) [57], while heatmaps were created using *Morpheus* (https://software.broadinstitute.org/morpheus/) [58], providing a clear visualization of gene expression patterns.

## Results

### Distinct expression patterns in samples with high and low tumor purity

**Fig. 1:**
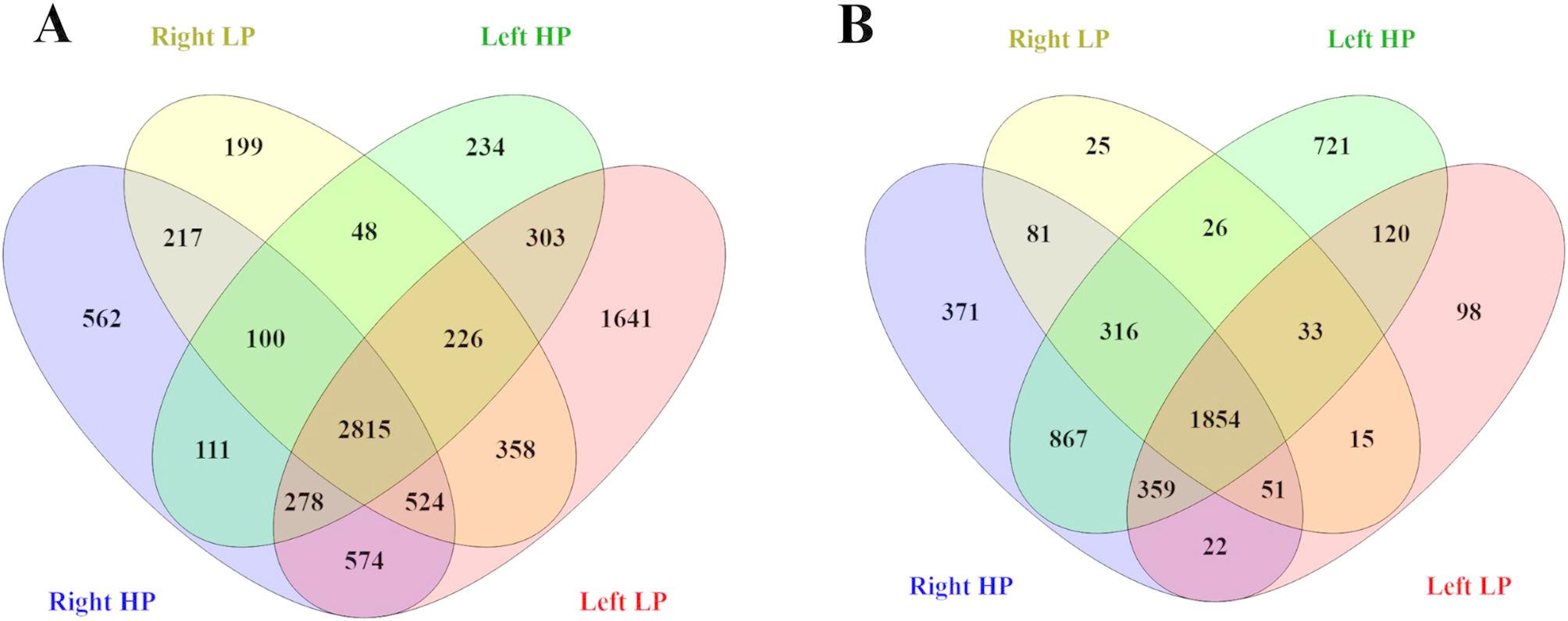
Differentially expressed genes in groups with varying levels of inflammatory infiltrate. Samples characterized by a high density of inflammatory cells were classified as low purity (LP), whereas those with a low density of inflammatory cells were classified as high purity (HP). **A** Upregulated genes identified across groups based on differential expression analysis. **B** Downregulated genes identified across the same comparisons. The interactome analysis between different anatomical regions revealed ligand–receptor pairs exhibiting distinct expression patterns across the regions analyzed

Differential expression analysis revealed molecular differences between intestinal regions in samples with varying tumor purity. The analysis identified differentially expressed genes within each group (Table S1, and Fig. S2). Among upregulated genes, 562 were exclusive to high-purity right-side samples, while 199 were unique to low-purity right-side samples. On the left side, 234 genes were found in high-purity samples, whereas 1,641 were specific to low-purity samples (Fig. 1A). A similar trend was observed for downregulated genes, with 371 exclusive to high-purity right-side samples and 25 unique to low-purity right-side samples. In the left-side samples, 721 genes were downregulated in high-purity cases, while 98 were specific to low-purity samples (Fig. 1B). Subsequently, the CMS classification of each sample was assessed. Samples originating from the right side were predominantly classified as CMS2, although right-sided high-purity (RHP) samples exhibited an equal distribution between CMS2 and CMS4. In contrast, left-sided samples were primarily classified as CMS4, regardless of tumor purity (Table S2 and Fig. S3).

### Interactome analysis across origin sites reveals ligand-receptor pairs with region-specific behaviors

**Fig. 2:**
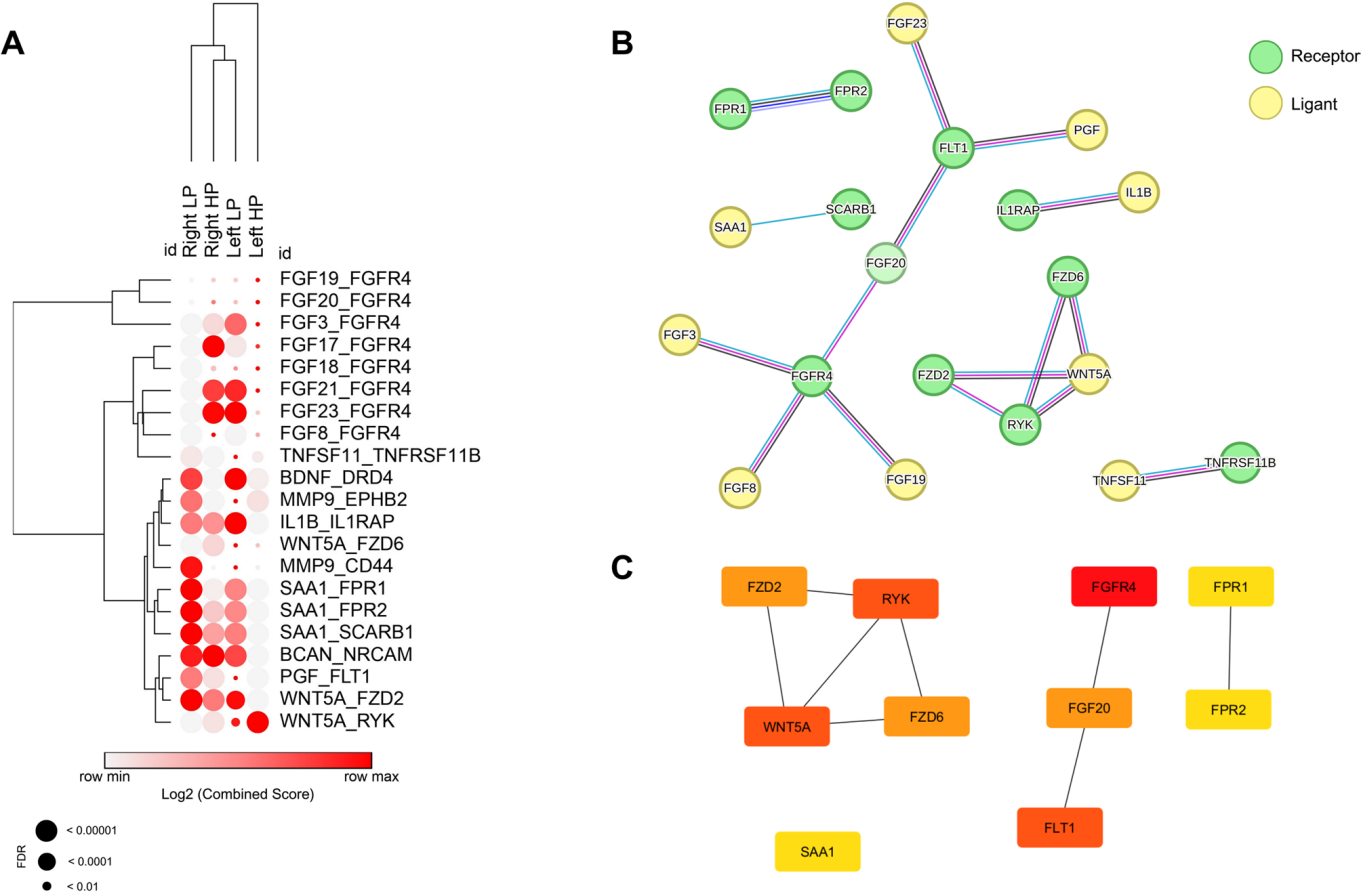
**A** Interactome pairs that were altered in specific components across various regions of the large intestine. **B** Protein-protein interaction network of the altered genes. **C** Top 10 genes with the highest number of interactions in the protein-protein network, identified using the degree method in the CytoHubba extension

After conducting differential expression analysis, upregulated genes were compared to the list of ligands and receptors provided by Ramilowski and colleagues [32]. Only significant ligand-receptor pairs, where both the ligand and receptor showed a substantial change (FC > 1.5 and FDR < 0.01) in at least one of the analyzed regions, were considered. This led to the identification of 21 interaction pairs (Fig. 2A), with the highest communication scores for both ligands and receptors, as well as Fold Change values (Table S2 and Fig. S3). A protein-protein interaction network was also constructed (Fig. 2B). Using the degree algorithm, we identified the top 10 genes with the most interactions: *FGFR4*, *FLT1*, *WNT5A*, *RYK*, *FGF20*, *FZD2*, *FZD6*, *FPR1*, *FPR2*, and *SAA1* (Table 2; Fig. 2C). The detected genes showed differential expression on the TNM Plot platform when comparing data from normal and tumor samples and in the different regions and CMS available (Table S3 and Figs. S4A-C, S5 and S6).

**Table 2:** Top 10 Protein-Protein interactions ranked by the degree algorithm

Rank	Gene	Score
1	*FGFR4*	4
2	*FLT1*	3
2	*WNT5A*	3
2	*RYK*	3
5	*FGF20*	2
5	*FZD2*	2
5	*FZD6*	2
8	*FPR1*	1
8	*FPR2*	1
8	*SAA1*	1

### Enrichment analysis identifies pathways related to cell proliferation, fibroblast communication, and the WNT pathway

After identifying the primary interaction pairs with the highest communication scores, the set of Ligands and receptors underwent functional enrichment analysis to explore the associated processes and pathways. We utilized the GO-Biological Process and KEGG 2021 Human datasets and obtained spreadsheets for each. Next, the enriched terms were separated for each ligand and receptor, selecting only those common to both within the pair (Table S3). This resulted in 33 enriched terms for the identified pairs.

**Fig. 3:**
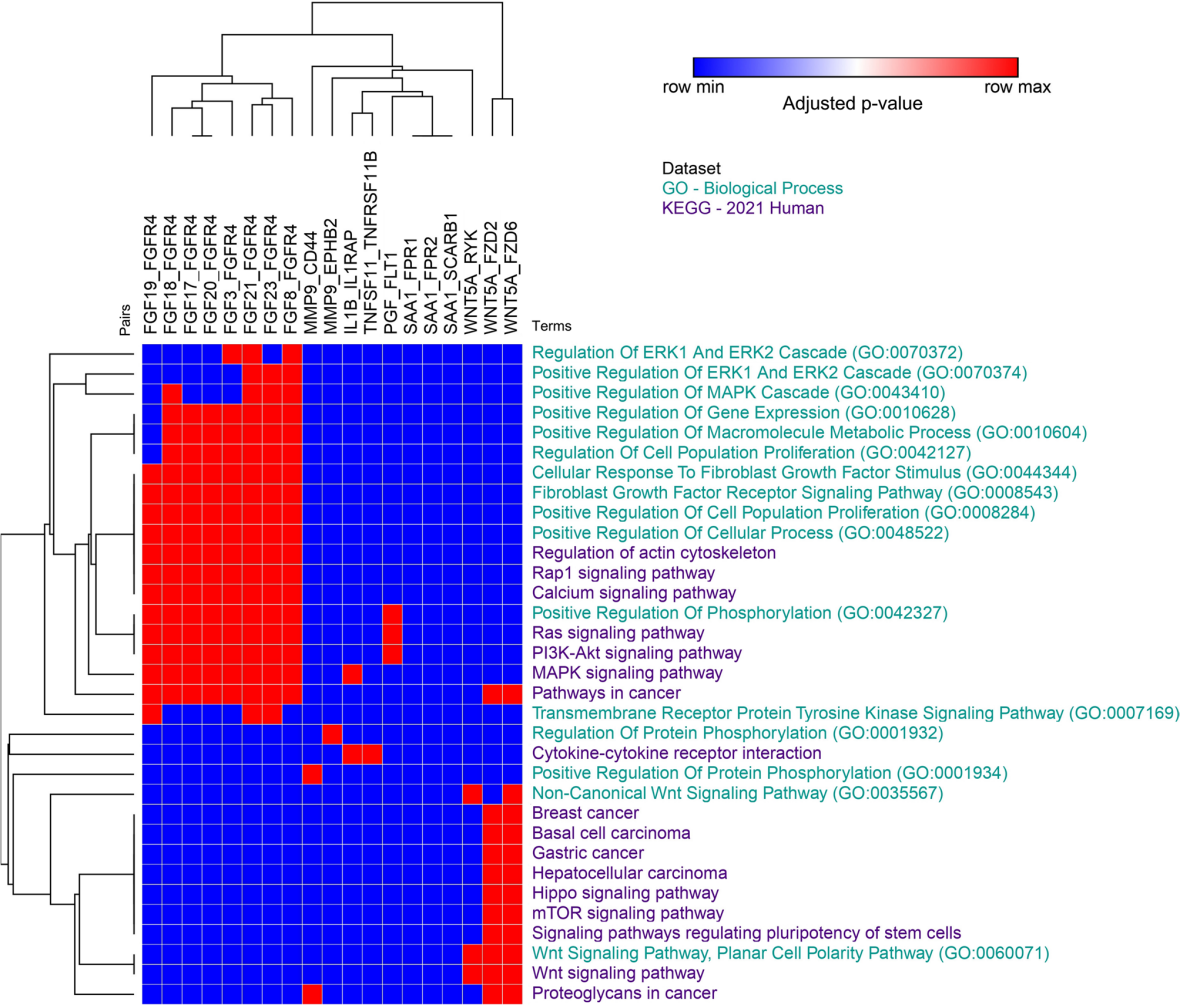
Pair enrichment heatmap generated using the EnrichR tool, based on GO-Biological Process and KEGG 2021 Human datasets. The rows and columns were clustered using Euclidean distance to evaluate the similarity between interaction pairs and enriched terms

After adjusting the *p*-value using the Euclidean distance algorithm, we observed clustering of terms related to two major processes: the Fibroblast Growth Factor (*FGF*) pathway and the *WNT pathway* (Fig. 3). Other notable enriched terms included pathways associated with cancer, such as “Pathways in Cancer” and “Proteoglycans in Cancer.” Additionally, pathways related to specific cancers, including Breast, Gastric, and Hepatocellular carcinoma, were enriched. Lastly, signal transduction pathways, which are active in various cancers, such as mTOR, Ras, MAPK, and PI3K-AKT, were also identified, all of which are associated with growth and cell proliferation.

### Survival curves illustrate distinct survival patterns for each origin site

**Fig. 4:**
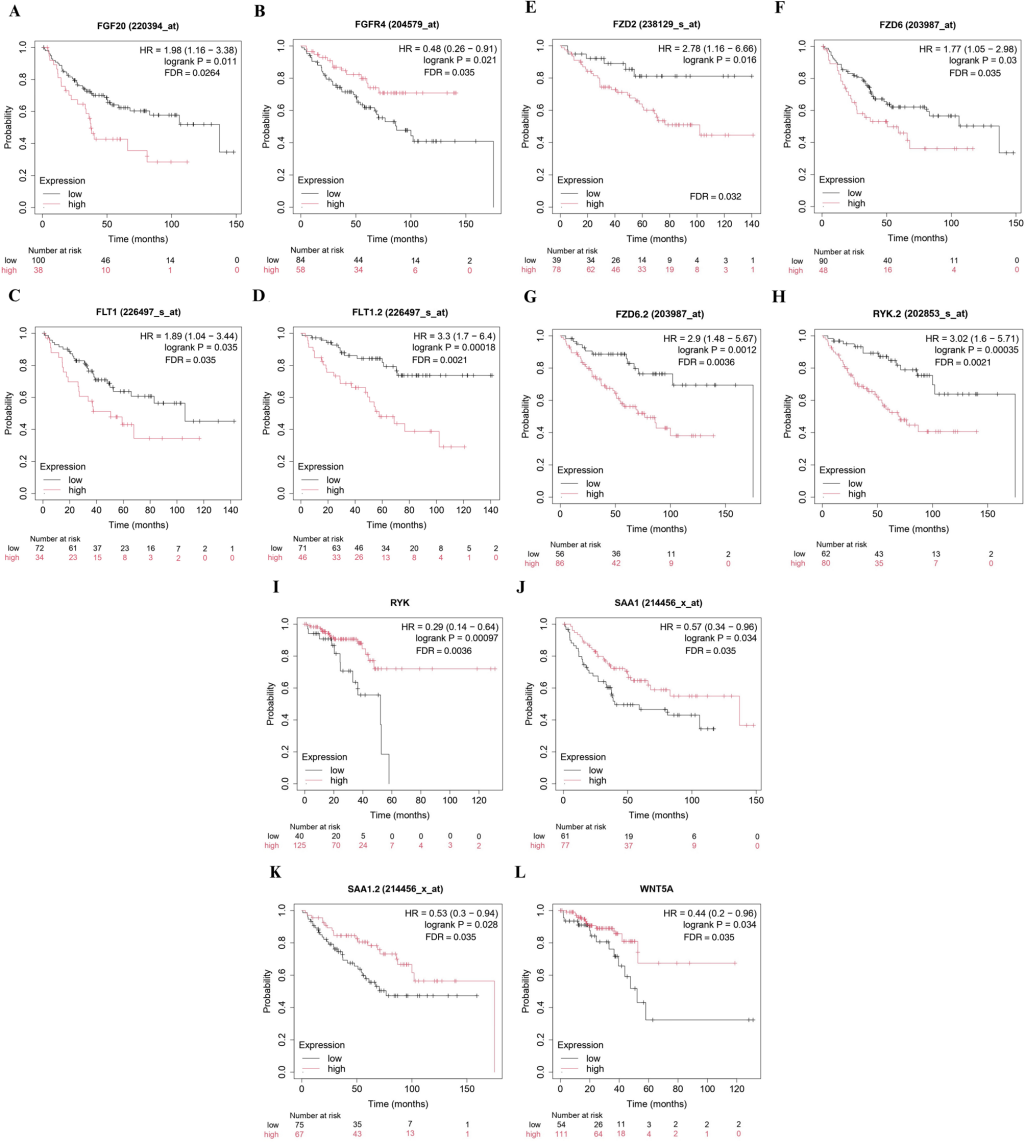
Survival curves for genes identified as key biomarkers in CRC tumorigenesis across different regions. Curves in red represent higher gene expression values, while those in black represent lower expression values. **A** Survival curve for the FGF20 gene in the right colon, showing a significant decrease in patient survival as gene expression increases (FDR = 0.0264). **B** Survival curve for the FGFR4 gene in the left colon, with a significant decrease in survival in patients with lower expression (FDR = 0.0.035). **C**-**D** Survival curves for the FLT1 gene in the right and left colon. In both cases, there is a significant reduction in survival for patients with higher expression (FDR = 0.035 and *p* = 0.0021, respectively). **E** Survival curve for the FZD2 gene in the left colon, showing a significant reduction in survival for patients with higher expression (*p* = 0.016). **F**-**G** Survival curves for the FZD6 gene in the right and left colon. Both show a reduction in survival for patients with increased expression (*p* = 0.035 and *p* = 0.0036, respectively). **H**-**I** Survival curves for the RYK gene in the left colon and rectum. A significant reduction in survival is observed in both regions, with high expression in the left colon (FDR = 0.0021) and low expression in the rectum (FDR = 0.0036). **J**-**K** Survival curves for the SAA1 gene in the right and left colon. Both show a reduction in survival for patients with low gene expression values (FDR = 0.035 and FDR = 0.035, respectively). **L** Survival curve for the WNT5a gene in the rectum, demonstrating a significant reduction in survival for patients with low expression values (FDR = 0.035)

When Subjected to Survival analysis, the 10 genes with the highest interaction scores exhibited distinct survival patterns based on the intestinal site. For *FGF20* (Fig. 4A), survival in the right colon showed a significant decline (*p*-value < 0.05), while no significant changes were observed in the left colon or rectum. For *FGFR4* (Fig. 4B), a significant reduction in survival was observed in the left colon, with lower survival linked to higher expression. *FLT1* showed significant survival reductions in both the right and left colon (Fig. 4C and D), but the *p*-value for the rectum was 0.073. Both *FPR1* and *FPR2* showed no significant differences in survival across any region or side. For *FZD2* (Fig. 4E), a significant survival reduction was observed only in the left colon with high expression.

In contrast, *FZD6* demonstrated a significant decrease in survival in both the right (*p* = 0.03) and left colon (*p* = 0.0012), but no significant change was noted in the rectum (Fig. 4F, G). *RYK* exhibited a significant reduction in survival in both the left colon and rectum, but the survival pattern differed: high expression in the left colon correlated with reduced survival, while in the rectum, the opposite pattern was observed (Fig. 4H, I). *SAA1* was associated with lower survival in the colon, with a significant survival reduction linked to low gene expression (Fig. 4J, K). Lastly, *WNT5A* showed reduced survival only in the rectum, which was also linked to lower gene expression (Fig. 4L).

### FZD2 exhibits differential sensitivity in rectal tumor cells and represents a potential biomarker

**Fig. 5:**
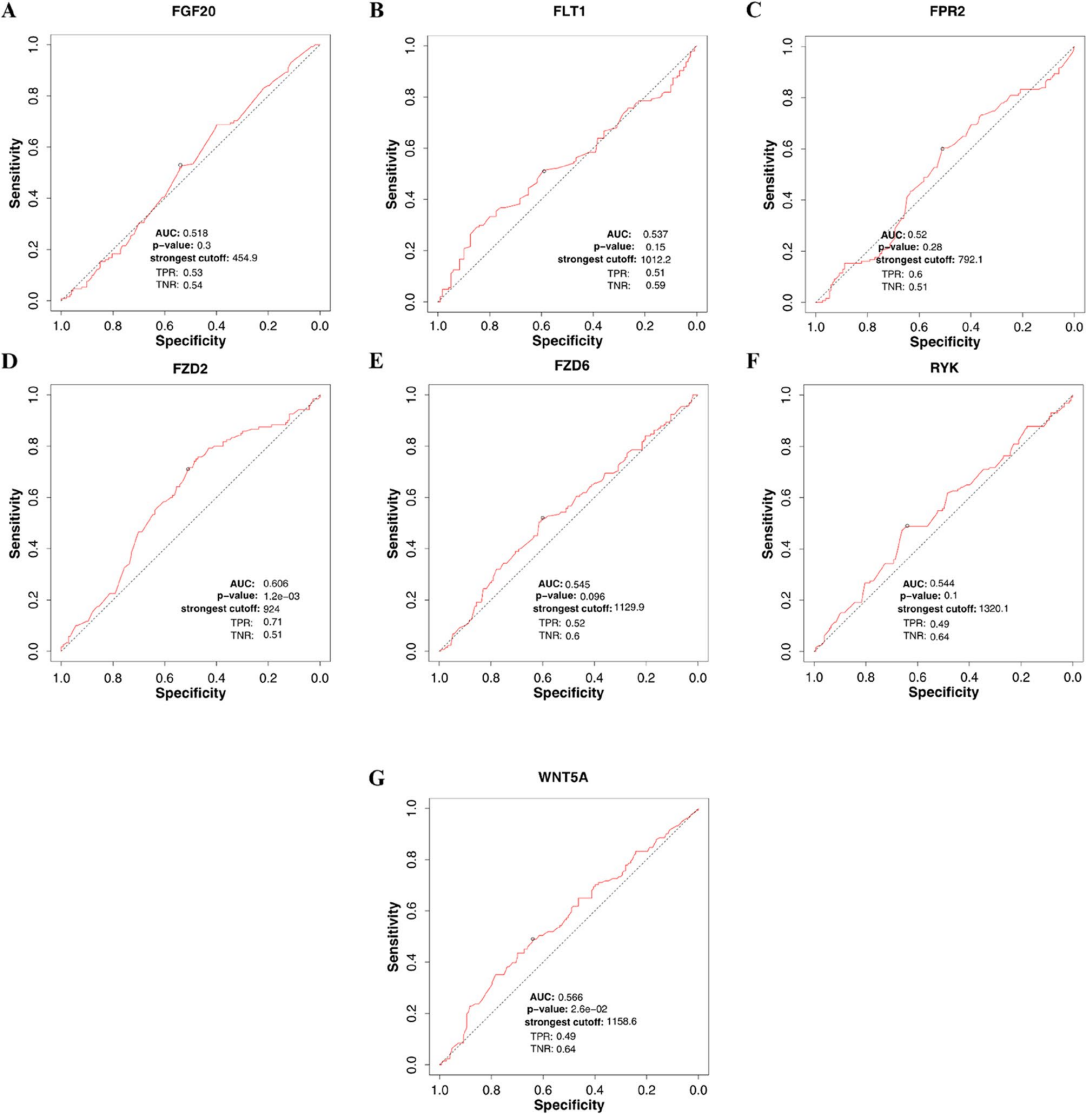
ROC curves for the markers identified in our analysis for the rectum, using the TNMplot tool. ROC curves for the markers identified in our analysis for the colon, using the TNMplot tool. In this region, only the FZD2 gene showed significant value as a predictive and therapeutic biomarker for this region **d**. FZD2 showed AUC = 0.606 and *p*-value = 0.0012, qualifying as a moderate biomarker

We assessed the predictive and diagnostic potential of the identified candidate genes across tumor regions using ROC curve analysis (Supplementary Table 6). In colon samples, none of the genes demonstrated statistically significant discriminatory power between therapy-sensitive and non-sensitive cells based on gene expression levels (Fig. S7). In contrast, in rectal samples, FZD2 showed a significant predictive value, with an area under the curve (AUC) of 0.606 and a *p*-value of 0.0012, suggesting its potential as a therapeutic biomarker specifically for rectal tumors (Fig. 5).

### scRNA-seq analysis reveals that genes with the highest degree values show altered expression across various cell types within the tumor microenvironment

One of the primary objectives of this interactome analysis is to assess the behavior of the Tumor Microenvironment (TME). Even with high-purity samples, marker expression can still be detected across various cell types within the tumor. Using the scRNA-seq study by Lee et al. [39], we were able to identify the sites where genes with the highest degree of expression were observed. Our analysis revealed eight distinct cell populations within the TME: T-cells, malignant cells, epithelial cells, macrophages, B-cells, fibroblasts, endothelial cells, and mast cells (Fig. S5).

**Fig. 6:**
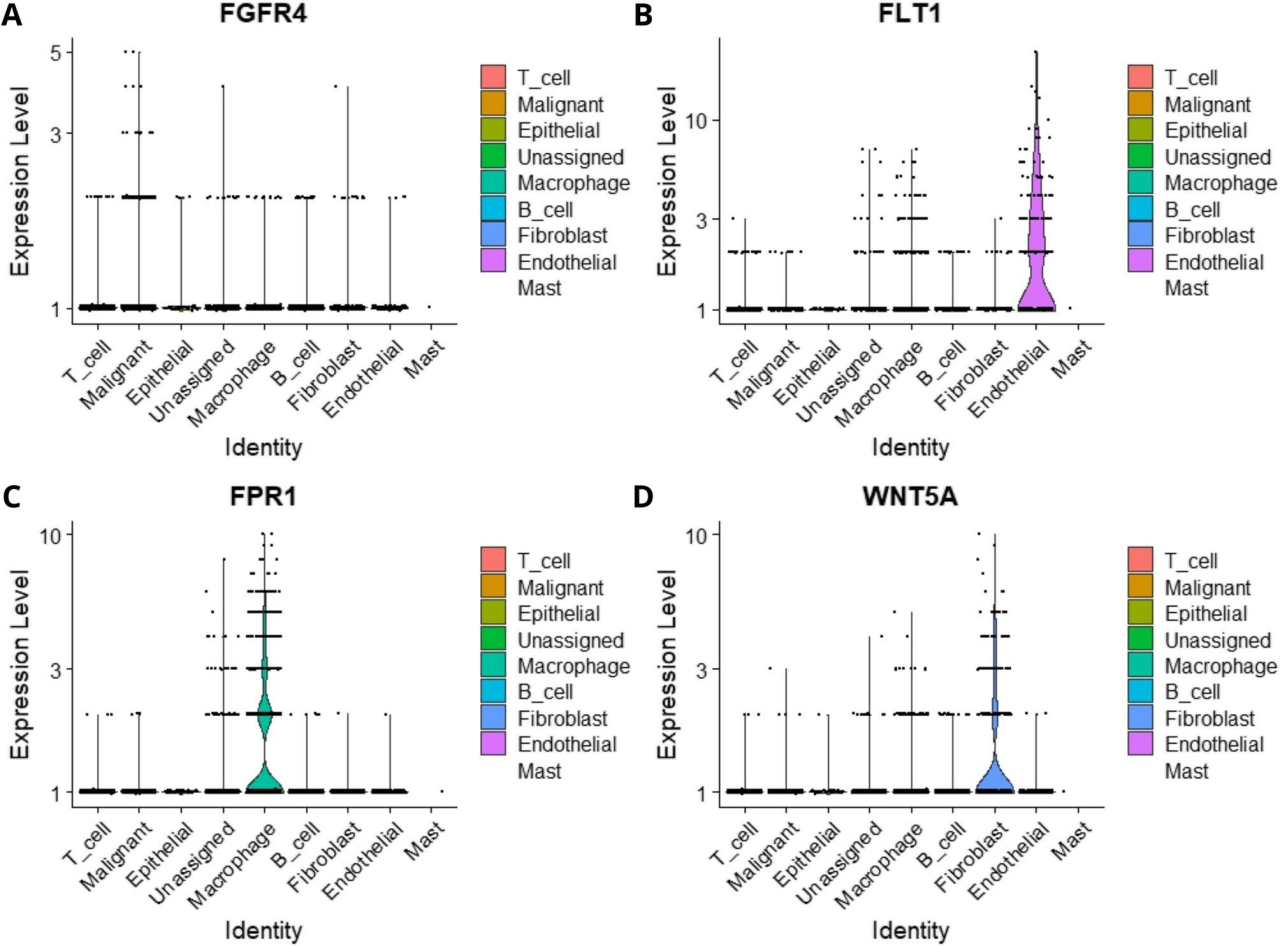
Expression levels of genes identified as key contributors to communication between cells and tumor microenvironment (TME), in the single cell RNA-seq study by Lee et al. 2020. **A***FGFR4* expression levels in CRC TME cells. **B***FLT1* expression levels in CRC TME cells, with higher expression observed in endothelial cells (pink). **C** FPR1 expression levels in CRC TME cells, showing higher expression in macrophages (light green). **D***WNT5a* expression levels in CRC TME cells, with greater expression observed in fibroblasts (light blue)

Among the 10 identified genes, four stood out for their expression in specific cell types within the TME (Fig. 6): *FGFR4*, *FLT1*, *FPR1*, and *WNT5A*. *FGFR4* exhibited higher expression in malignant and epithelial cells (Fig. 6A). *FLT1* showed high expression in endothelial cells (Fig. 6B). In contrast, *FPR1* was significantly expressed in macrophages (Fig. 6C). Lastly, *WNT5A* was predominantly expressed in fibroblasts (Fig. 6D). These findings suggest that genes associated with tumor carcinogenesis may not be highly expressed in the cancer cells themselves, but rather in tumor-associated cells such as tumor-associated macrophages (TAMs) and cancer-associated fibroblasts (CAFs).

### FGFR4 is a potential target for Pharmacological repositioning in CRC treatment

**Fig. 7:**
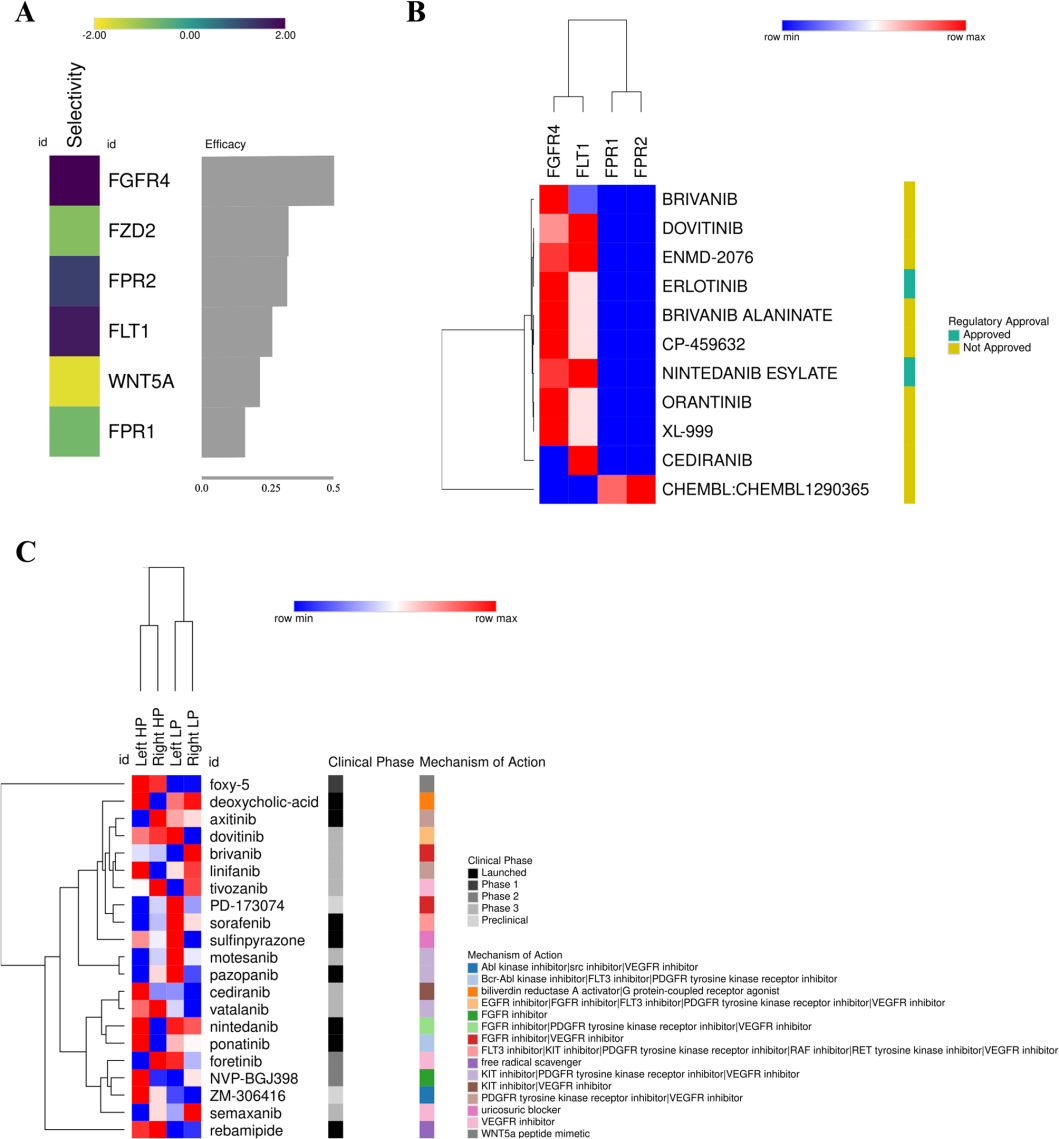
Pharmacological prediction of genes identified as potential biomarkers and therapeutic targets for CRC carcinogenesis: **A** Selectivity and efficacy of targets analyzed using the shinyDeepMap tool. *FGFR4*, *FPR2*, and *FLT1* demonstrated positive selectivity and efficacy, highlighting them as potential biomarkers and therapeutic targets for CRC. **B** Predicted drugs capable of interacting with the identified CRC biomarker candidates, based on interaction scores from the DGIDB platform. Blue values represent lower gene-drug interaction scores, while red values indicate higher scores. The regulatory status of the drugs is also shown: approved drugs are marked in green, while those in clinical trials are marked in orange. **C** Pharmacological predictions based on gene expression across different sites in the large intestine, analyzed using the OCTAD platform. Blue values indicate lower interaction scores, while red values signify higher scores. The clinical development stages of the drugs are represented in shades of gray, while various colors indicate the described mechanisms of action

The genes identified as potential biomarkers for CRC progression, categorized by site, were analyzed using the shinyDeepMap platform. This analysis aimed to determine which genes are highly druggable, focusing on their efficacy and selectivity. *FGFR4* showed the highest efficacy and selectivity, highlighting its potential as a pharmacological target for drugs designed to impair tumor progression. *FPR2* and *FLT1* also exhibited favorable selectivity, suggesting these genes may be promising targets for therapeutic interventions in CRC alongside *FGFR4* (Fig. 7A). Subsequently, the top 10 genes ranked by degree were submitted to the DGIDB platform to identify potential drugs targeting these genes. Once again, *FGFR4* stood out, with numerous related drugs and the highest interaction scores. The *FPR1* and *FPR2* genes interacted with only one drug each. Additionally, *FLT1* showed high interaction values with four drugs in the database, three of which are predicted to be potential *FGFR4* inhibitors (Dovitinib, ENMD-2076, and Nintedanib Esylate), positioning them as potential CRC treatments targeting multiple genes.

When using the OCTAD platform to analyze drug behavior based on intestinal sites, we found a significant presence of various FGFR4 inhibitors. However, the signature values for these inhibitors varied across different regions of the intestine, suggesting that drug effectiveness and responses may differ depending on the tumor’s origin. Thus, FGFR4 is a potential pharmacological target for treating CRC, but its response to drugs may vary by tumor site. Additionally, some drugs targeting FGFR4 also interact with other key genes identified in this analysis, such as FLT1. Our analysis using the OCTAD platform also identified WNT5A as another potential target. Notably, Foxy-5 appeared as a promising drug for targeting WNT5A, with its influence being particularly pronounced in samples with high tumor purity, indicating its potential effectiveness in such contexts.

To explore drugs likely to reverse the expression profiles in samples from different regions with varying tumor purities, we utilized the L1000FWD tool (Table S6). A Venn diagram (Fig. S6) was constructed to identify Drugs exclusive to each region. The analysis revealed the following drug exclusivities: 25 Drugs exclusive to the right region with high tumor purity, 24 Drugs exclusive to the right region with low tumor purity, 38 Drugs exclusive to the left region with high tumor purity, and 37 drugs exclusive to the left region with low tumor purity. This information underscores the distinct therapeutic options available based on tumor purity and regional differences.

## Discussion

Colorectal cancer (CRC) has three main lines of treatment, which are administered based on the identification of specific tumor markers. The first line of treatment involves fluoropyrimidines, which act as DNA antimetabolites, disrupting DNA synthesis. Commonly used drugs in this category include Fluorouracil and Irinotecan, often used alone or in combination with VEGF or EGFR inhibitors [50]. However, despite these combinations, tumors often develop resistance to treatment. This article proposes a novel approach to identifying potential diagnostic, prognostic, and therapeutic biomarkers for CRC. The focus is on genes whose final protein products are located in extracellular regions or outside the cytoplasm—referred to as the secretome. Since these proteins are secreted, they can be detected non-invasively in blood or feces [8].

By integrating bulk-RNA-seq data with secretome data, we developed an approach that enabled us to infer the process of cellular interaction in CRC through the construction of an interactome. This analysis revealed the presence of distinct molecular markers depending on the tumor’s site of origin. We identified *FGFR4*, *FLT1*, and *WNT5A* as potential biomarkers for CRC, as they exhibited differential expression between sites, formed significant interactions with other genes, and influenced patient survival based on the tumor’s origin. Through drug repositioning using computational biology, we identified Dovitinib and Nintedanib as potential candidates for CRC treatment, as these drugs inhibit pathways associated with the identified biomarkers.

New lines of therapy have emerged, combining traditional drugs with others targeting molecular markers to overcome treatment resistance [59, 60]. Technological advancements in sequencing techniques have improved our understanding of the molecular markers involved in tumor progression. These markers are crucial for the effectiveness of both classic and emerging treatments, such as immunotherapy [7, 60]. Our analysis highlighted the significant role of *FGFR4* in various regions. *FGFR4* encodes a receptor tyrosine kinase involved in key processes like cell proliferation, migration, and differentiation [61]. Although its role in CRC has been less studied, existing research indicates its involvement in critical tumor progression processes, such as epithelial-mesenchymal transition, cell proliferation, and tumor cell migration [62]. These processes are linked to the modulation of fundamental pathways, including JAK/STAT, PI3K/AKT, and Wnt pathways, which are essential for cell proliferation. Mutations in *FGFR4* have been shown to act as an oncogene in rectal tumors, accelerating tumor development [61, 62]. Consequently, selective FGFR4 inhibitors have been developed to hinder tumor progression, resulting in significant reductions in migration, invasion, and epithelial-mesenchymal transition [63–69].

*FLT1* (VEGFR1) is another gene that plays a pivotal role in tumor development, primarily through its involvement in angiogenesis, tumor growth, and metastatic progression [70]. This gene encodes the vascular endothelial growth factor (VEGF), a tyrosine kinase receptor found in endothelial cells that promotes blood vessel formation [71, 72]. *FLT1* also contributes to resistance against certain drugs during tumor development and progression. For instance, in breast cancer, high expression of *FLT1* has been linked to resistance to poly(ADP-ribose) polymerases, enzymes that induce DNA damage in cells with high expression of the BRCA1 and BRCA2 oncogenes [72]. Moreover, *FLT1* is involved in the progression of various tumors, as it activates the WNT β-catenin pathway, which plays a critical role in glioma development. This same pathway is also associated with colon cancer, and one of the markers identified in our analysis was *WNT5A*, which also activates this pathway [73].

In CRC, few studies have exclusively focused on the effects of *FLT1* on carcinogenesis. However, an increase in its expression has been observed in both primary and metastatic CRC tumor tissues compared to normal tissue. Notably, the expression level of *FLT1* varies with the disease stage, becoming significantly higher in stage IV [74]. Additionally, this variation in expression impacts serum *FLT1* levels, positioning this gene as a potential prognostic and diagnostic marker for CRC [75, 76]. Furthermore, *FLT1* has been linked to resistance to bevacizumab, as its expression is associated with *VEGFA* and *KDR* [76].

Although *FLT1* plays a central role in regulating the carcinogenic process, its relative expression remained similar and non-significant across all intestinal sites (Fig. S3). Consequently, we decided to evaluate the expression of *PGF*, a ligand that exhibited differential expression in low-purity samples from both sites, unlike in high-purity samples. *PGF*, a member of the *VEGF* family, is associated with key processes involved in tumor progression, such as angiogenesis, cell proliferation, and migration [77]. Moreover, with the development of drugs targeting the *VEGF-A* receptor, *PGF* has gained attention for its role in pharmacological resistance to bevacizumab [78]. Therefore, *PGF* is strongly associated with various cells in the tumor microenvironment (TME), modulating tumor cells and creating an environment conducive to carcinogenesis [78].

In CRC, studies have suggested that *PGF* could serve as a marker for tumor malignancy. One of its key functions in this type of tumor is its direct relationship with angiogenesis, as it is regulated by *WNT2*. The joint expression of *PGF* and *WNT2* has been linked to increased vessel formation in xenograft models of CRC [79]. Additionally, an analysis of TCGA patients, combined with scRNA-seq data, revealed that higher expression of *PGF* correlates with lower survival rates, further supporting its potential as a biomarker and therapeutic target [80]. This led to the development of aflibercept [81], a selective inhibitor of *VEGF-A* and *VEGF-B*, which is currently used as a second-line treatment for CRC with demonstrated efficacy. However, our analysis indicates that *PGF* is associated with lower-purity samples, suggesting significant communication between TME cells and tumor cells [82].

*WNT5a*, unlike the other genes discussed, is a well-established biomarker for various tumor types due to its significant role in activating the non-canonical WNT pathway [83]. In cancer, *WNT5a* exhibits a dual role, showing both oncogenic and anti-tumor activities [84]. This is also true for CRC, where *WNT5a* has been implicated in regulating processes such as cell division, progression, adhesion, and polarization. However, the effect of *WNT5a* in CRC is dependent on the mode of activation of the WNT pathway, as different activation mechanisms can either positively or negatively regulate these processes [85, 86]. The influence of *WNT5a* on CRC is further shaped by the various factors that impact its expression, as multiple stimuli can modulate this pathway [86]. By utilizing the interactome to evaluate potential biomarkers, we highlighted the complexity of the interactions involving *WNT5a*. Our analysis revealed an increase in WNT5a and its receptor *FZD2*, which is associated with oncogenic activity [87]. Notably, the highest combined interaction scores for this pair were found in low-purity samples, suggesting a greater inflammatory infiltrate. This is particularly significant because *TAM*-induced activation of this interaction also contributes to its oncogenic effect [88].

Elevated expression of ligand–receptor pairs was observed in stromal cells within the TME, underscoring the active involvement of non-malignant cellular components in colorectal cancer progression. This observation is critical, as previous studies have demonstrated that these stromal cells significantly influence tumor progression and thus represent potential therapeutic targets [89]. In our analysis, fibroblasts, macrophages, and endothelial cells were identified as the primary cell populations expressing the proposed markers.

Fibroblasts demonstrated elevated expression of WNT5a, a key ligand in the WNT signaling pathway, which is widely recognized for its critical role in various aspects of CRC tumorigenesis [90]. Within the tumor microenvironment, cancer-associated fibroblasts (CAFs) are prominent stromal components that actively contribute to carcinogenesis by modulating the tumor stroma and regulating local energy metabolism. Notably, CAFs influence glucose metabolism through upregulation of GLUT-1 expression. Additionally, enhanced glutamine and fatty acid metabolism by CAFs impacts apoptosis and promotes tumor cell migration [91, 92].

Macrophages were another cell population prominently expressing the FPR1 receptor. As previously noted, these cells can differentiate into TAMs, which play key roles in processes such as tumor proliferation, migration, and angiogenesis [93]. Accordingly, therapeutic strategies targeting the TME in CRC are currently under investigation, primarily focusing on modulating the metabolic interactions within the TME to attenuate carcinogenesis. Most compounds tested to date act by disrupting metabolic pathways, including glucose uptake, the tricarboxylic acid (Krebs) cycle, and fatty acid metabolism [91, 94]. In contrast, our study proposes an alternative approach aimed at mitigating molecular dysregulation in cellular regulatory processes. Consequently, Dovitinib and Nintedanib have been identified as promising candidate therapies for further evaluation.

Dovitinib emerged as the drug with the most significant interactions with genes when examining the intersection between pharmacological prediction datasets. This drug is a selective inhibitor of *VEGF*, *FGF*, and *PDGF* receptors, administered orally for tumors that have shown resistance to therapies targeting the same mechanisms. Dovitinib has been used, either alone or in combination with other medications, in cancers such as triple-negative breast cancer, gastric cancer, and endometrial cancer [95]. Numerous studies have demonstrated the effectiveness of Dovitinib, both as a monotherapy and in combination treatments. In triple-negative breast cancer, its combination with calcitriol has shown synergistic effects by reducing critical factors in tumor progression, including angiogenesis and cell division. Furthermore, this combination proved more effective at inducing DNA damage, as many cells exhibited halted division and aberrant morphologies, including multinucleation [96].

In addition to synergizing with natural molecules, Dovitinib can also enhance the efficacy of other chemotherapy drugs. A notable example is in the treatment of gastric cancer, where Dovitinib, when combined with nab-paclitaxel, showed an additive effect, leading to more significant inhibition of cell proliferation and angiogenesis [97]. While limited studies have evaluated the role of Dovitinib in CRC, it has been shown to exert its effects independently of *KRAS* or *BRAF* mutation status. The drug’s efficacy was observed across various cell lineages, even with differing mutations in these genes. It was found to decrease both cell proliferation and angiogenesis, although the results differed between in vivo and in vitro models [98].

Recent studies investigating the use of Dovitinib in CRC have reported conflicting outcomes in both in vitro and clinical settings. In CRC cell lines, combination treatment with dovitinib and oxaliplatin demonstrated a synergistic effect, significantly reducing cell viability and migration, increasing DNA damage, and upregulating apoptotic markers. These effects were also recapitulated in xenograft models, where combined therapy led to decreased tumor viability [99]. Conversely, a clinical study involving patients with colorectal and lung cancers — both resistant to anti-VEGF therapies — evaluated the efficacy of dovitinib addition. This study found no significant impact on tumor progression or on the expression of proangiogenic factors within the same gene family. Moreover, dovitinib administration was associated with increased adverse effects in patients [100]. Therefore, our study, which highlights Dovitinib as a potential candidate, emphasizes the need for further research to better understand its therapeutic role in CRC, particularly in inhibiting carcinogenesis-related signaling pathways and limiting tumor progression.

Finally, our study identified Nintedanib as a highly promising treatment for CRC. Originally developed for pulmonary fibrosis, Nintedanib is a potent selective inhibitor targeting crucial tyrosine kinases such as *VEGFR*, *FGF*, *PDGFR*, *SRC*, and *FLT-3* [101, 102]. By targeting these receptors, Nintedanib inhibits cell proliferation, particularly in cells involved in angiogenesis. It is currently used in combination with docetaxel for treating non-small cell lung carcinoma, showing relative efficacy and a favorable profile due to its minimal drug-drug interactions, limited influence by ethnicity and gender, and efficient excretion [103]. For CRC, antiangiogenic drugs like bevacizumab, ramucirumab, and regorafenib are often combined with standard chemotherapy. Nintedanib has shown similar efficacy to these drugs, with comparable progression-free survival (PFS). A key advantage of Nintedanib is that it targets more than just *VEGF* and its receptor, reducing the likelihood of chemoresistance [85]. Therefore, it is crucial to explore the biomarkers that correlate with the drug’s effectiveness in treating CRC [104]. Similar findings have been reported for other angiogenesis inhibitors like bevacizumab, which proved more effective in high-MSI and CMS1 tumors [105].

Several reviews have synthesized clinical studies at various stages that evaluated the efficacy of Nintedanib in CRC. Given the resistance of CRC to conventional anti-angiogenic therapies, Nintedanib presents a promising alternative due to its multi-targeted inhibition of the VEGF signaling pathway. Its therapeutic outcomes have been comparable to those of currently approved inhibitors; however, clinical trials specifically addressing tumors resistant to existing anti-VEGF treatments are still lacking. Further investigation is warranted, as preliminary clinical data have reported adverse effects associated with Nintedanib use in CRC patients, including diarrhea, stomatitis, and neutropenia [106–108]. Despite these challenges, repositioning Nintedanib as a treatment for CRC underscores the importance of this approach in clinical practice, as it involves drugs that already have clinical approval, simplifying the regulatory process for use in other cancers [109].

## Conclusion

CRC displays considerable molecular heterogeneity, underscoring the urgent need for enhanced early detection strategies. Liquid biopsy, based on circulating molecular markers, represents a promising approach for non-invasive diagnosis. In this study, we identified FGFR4, FLT1, WNT5A, and FZD2 as potential biomarkers for CRC, providing avenues for targeted therapeutic intervention. Repurposed drugs such as Dovitinib and Nintedanib demonstrate potential in inhibiting critical pathways involved in CRC progression, including cell proliferation and angiogenesis, thereby facilitating accelerated clinical translation through computational analyses. Nonetheless, the conclusions are constrained by the absence of experimental validation. Future investigations should incorporate comprehensive in vitro and in vivo studies to substantiate the diagnostic and therapeutic efficacy of these candidate genes and drugs. Such validation is essential to elucidate their mechanistic roles and to advance their clinical application in CRC management [99, 109–112].

## Supplementary Information

Supplementary Material 1.

## Data Availability

No datasets were generated or analysed during the current study.
