# Supplementary material for: Integrating the secretome and interactome to identify novel biomarkers and therapeutic targets in colorectal cancer
Source: Cell Commun Signal. 2025 Oct 10;23:428. doi: 10.1186/s12964-025-02424-4 (PMC12512465; doi:10.1186/s12964-025-02424-4)
Supplement: Supplementary file 1 — Supplementary Material 1. [file 12964_2025_2424_MOESM1_ESM.zip › Supplementary/Supplementary Data.docx]

**Supplementary Data**


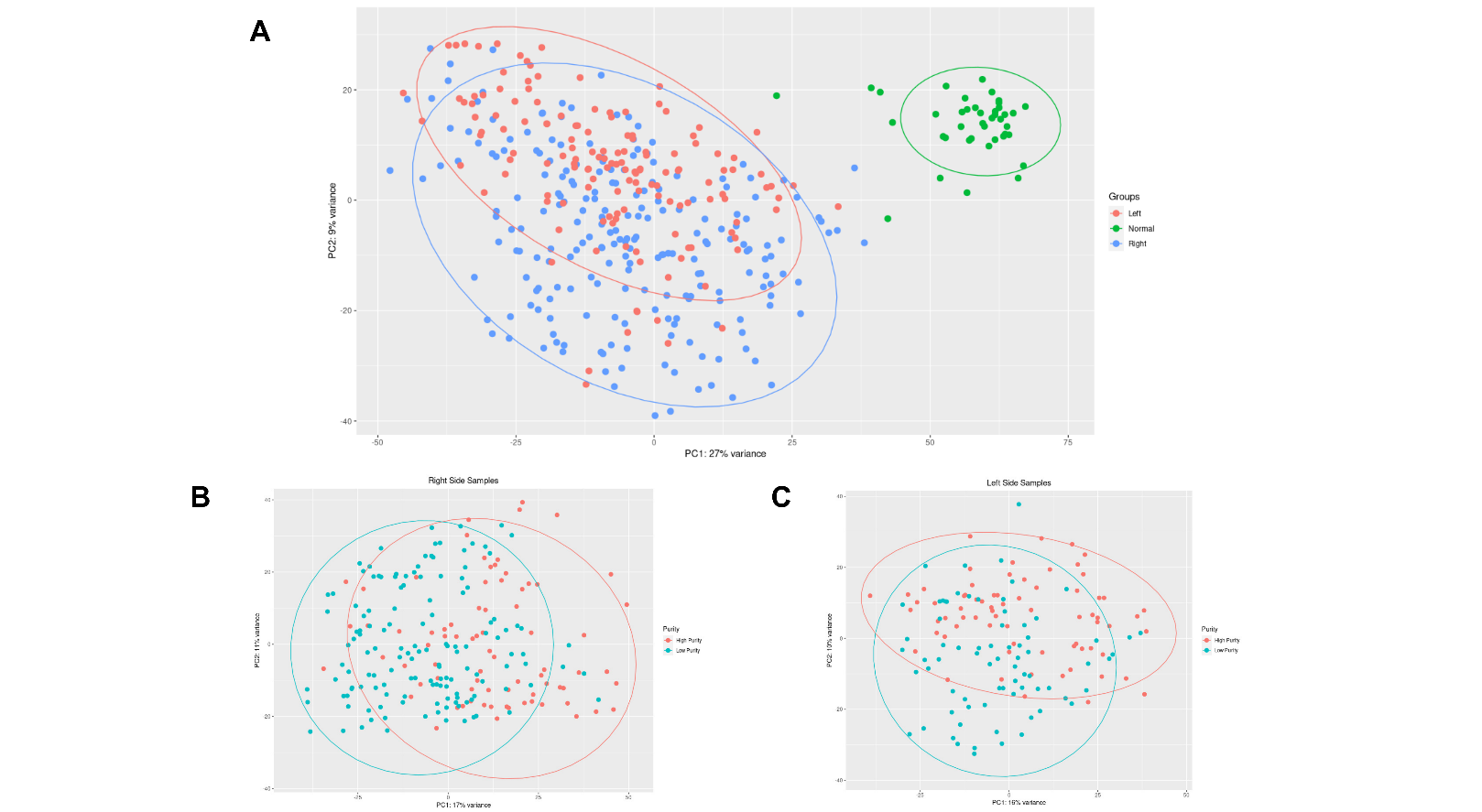


Supplementary Material 1: Supplementary Fig. 1: Principal Component Analysis (PCA) of COAD samples downloaded from the TCGA database. A PCA analysis of normal and tumor samples separated by region of the large intestine. Green represents the normal samples, red indicates tumor samples from the left region, and blue represents tumor samples from the right region. B PCA analysis of tumor samples from the right side of the large intestine, separated by purity. Red represents high-purity samples, and blue represents low-purity samples. C PCA analysis of tumor samples from the left side of the large intestine, separated by purity. Red represents high-purity samples, and blue represents low-purity samples.


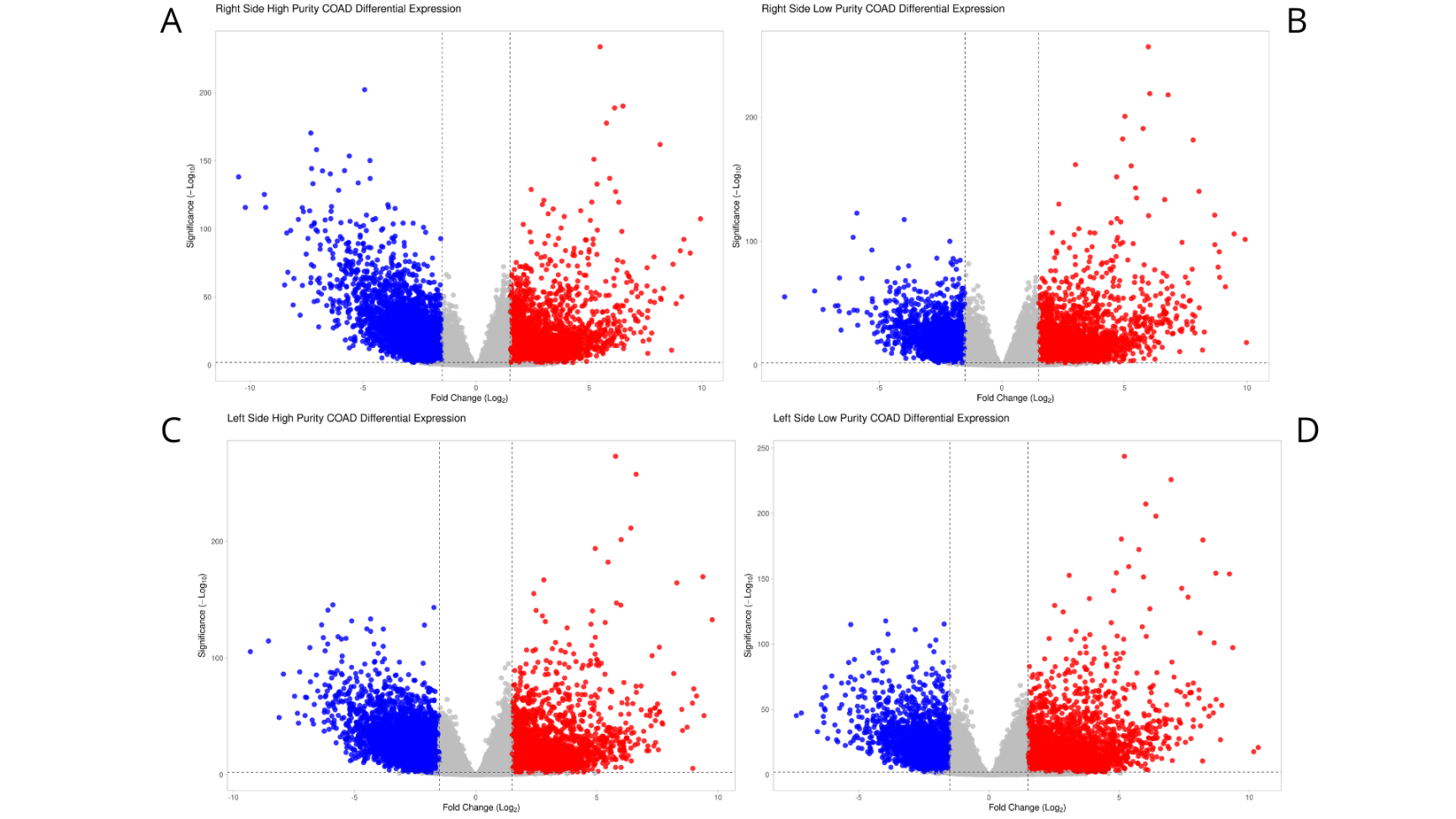


Supplementary Fig. 2: Volcano plots of differential expression analyses. Downregulated genes are shown in blue, and upregulated genes in red. A Volcano plot of the differential expression analysis comparing normal samples with high-purity large intestine tumor samples from the right region. B Volcano plot of the differential expression analysis comparing normal samples with low-purity large intestine tumor samples from the right region. C Volcano plot of the differential expression analysis comparing normal samples with high-purity large intestine tumor samples from the left region. D Volcano plot of the differential expression analysis comparing normal samples with low-purity large intestine tumor samples from the left region.


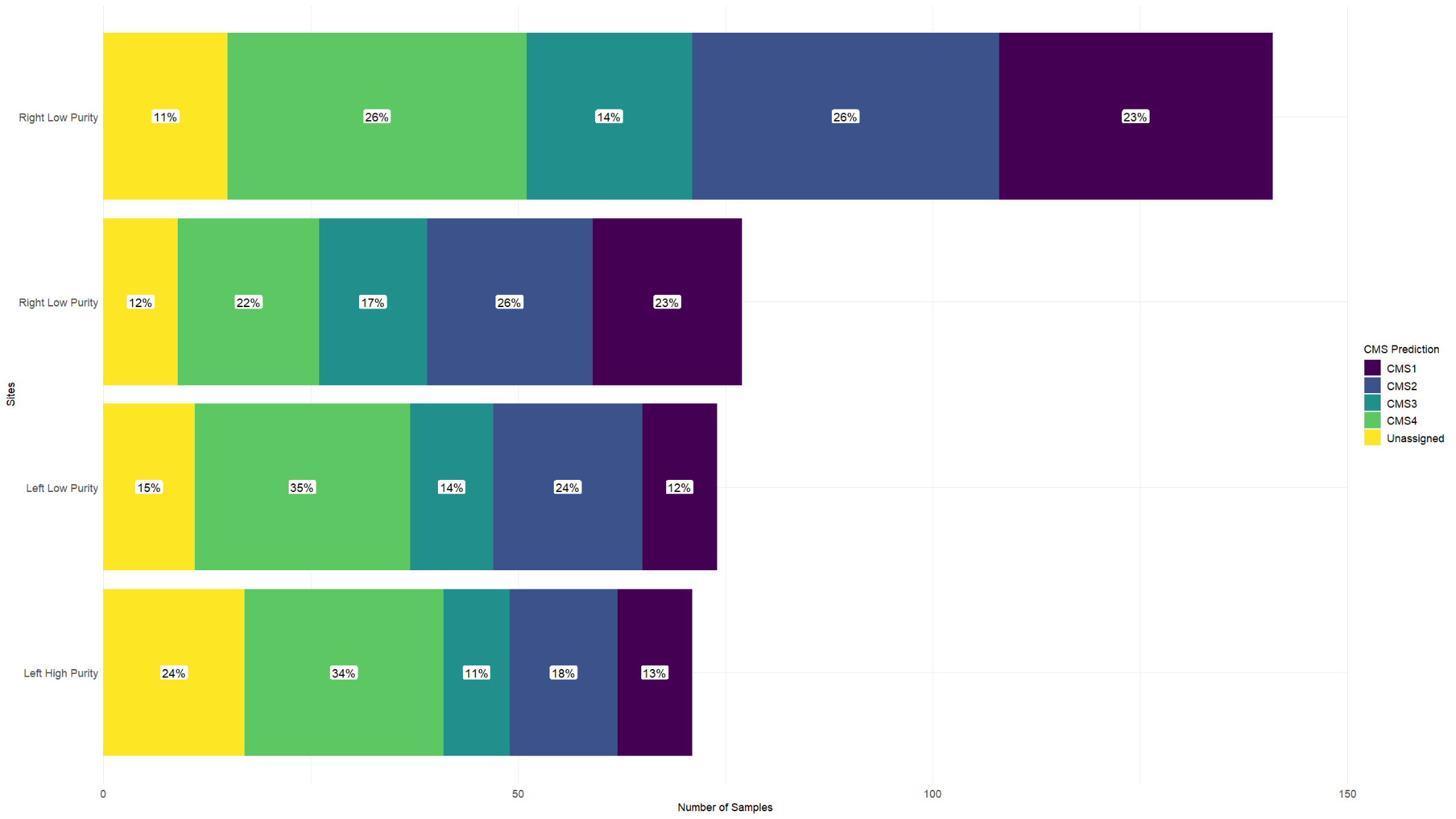


Supplementary Fig.3: Proportions of each CMS for the different sites and purities of the samples in the TCGA-COAD dataset. The samples were classified using CMScaller. CMS 1 samples are in purple, CMS 2 are in blue, CMS 3 are in olive green, and CMS 4 are in green. Samples that were not identified in any CMS are in yellow
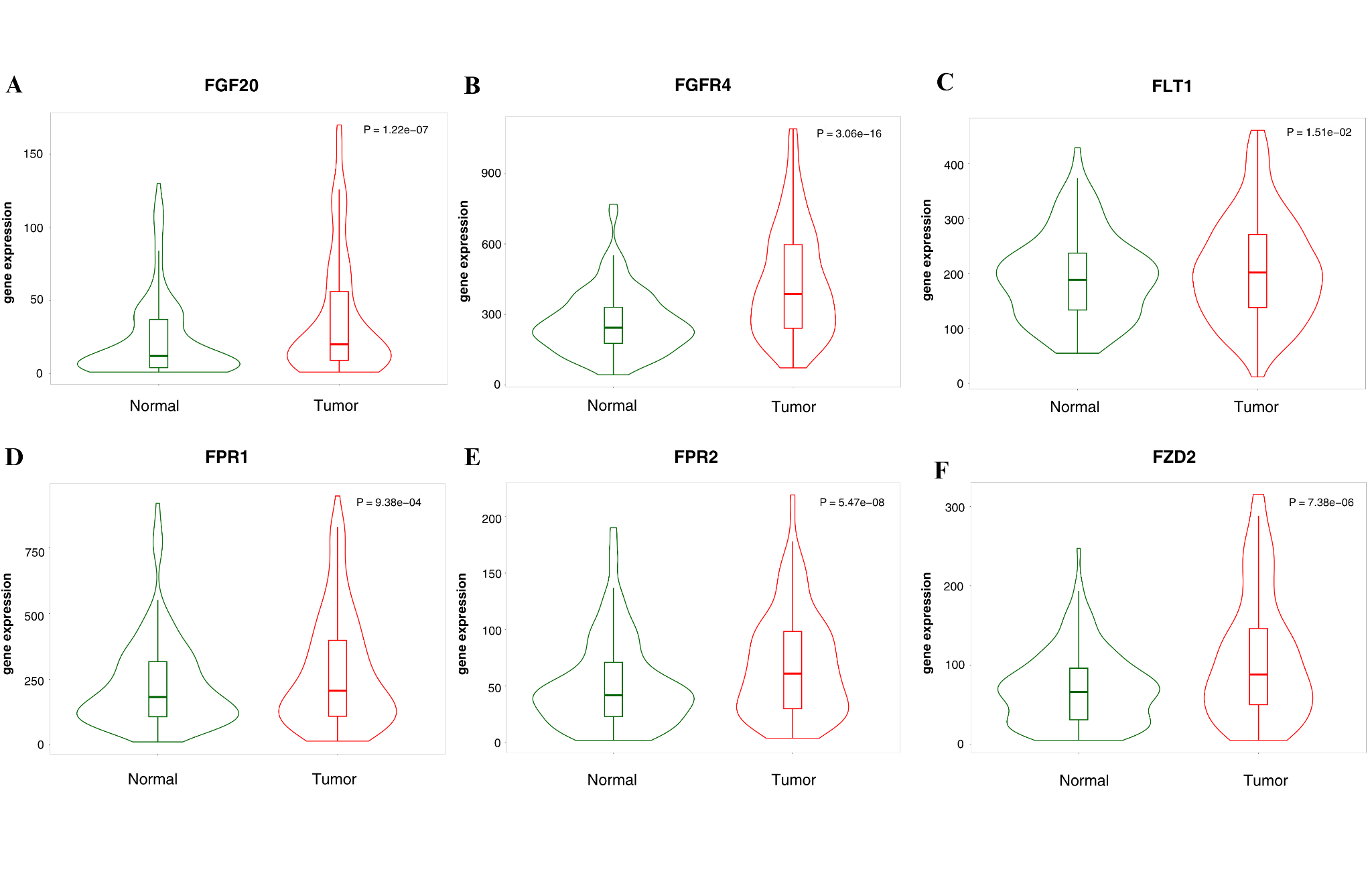

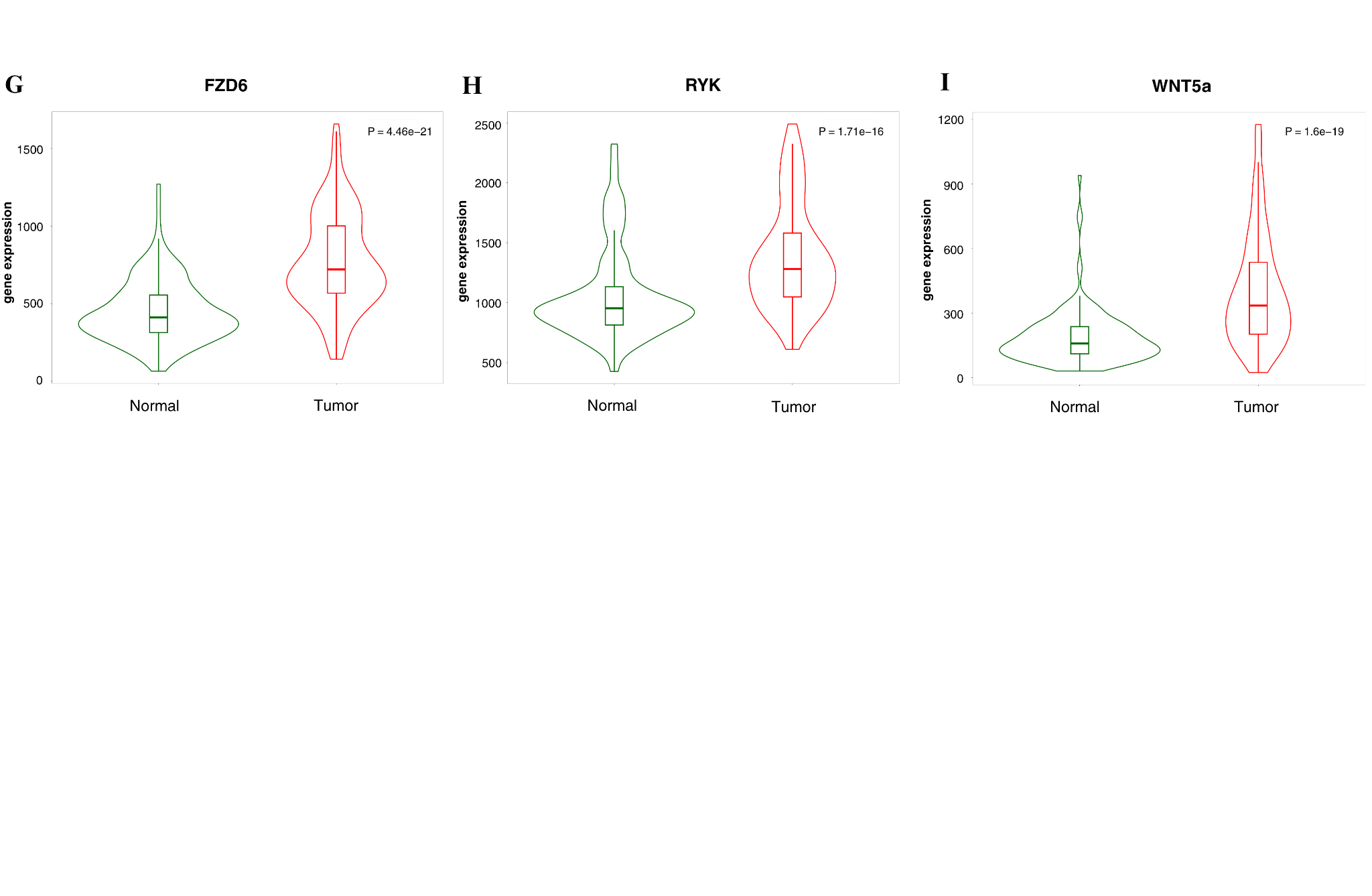


Supplementary fig 4a: Violin plot of the expression of potential biomarkers detected in our analysis in the Chip-Seq database in the TNMplot tool.


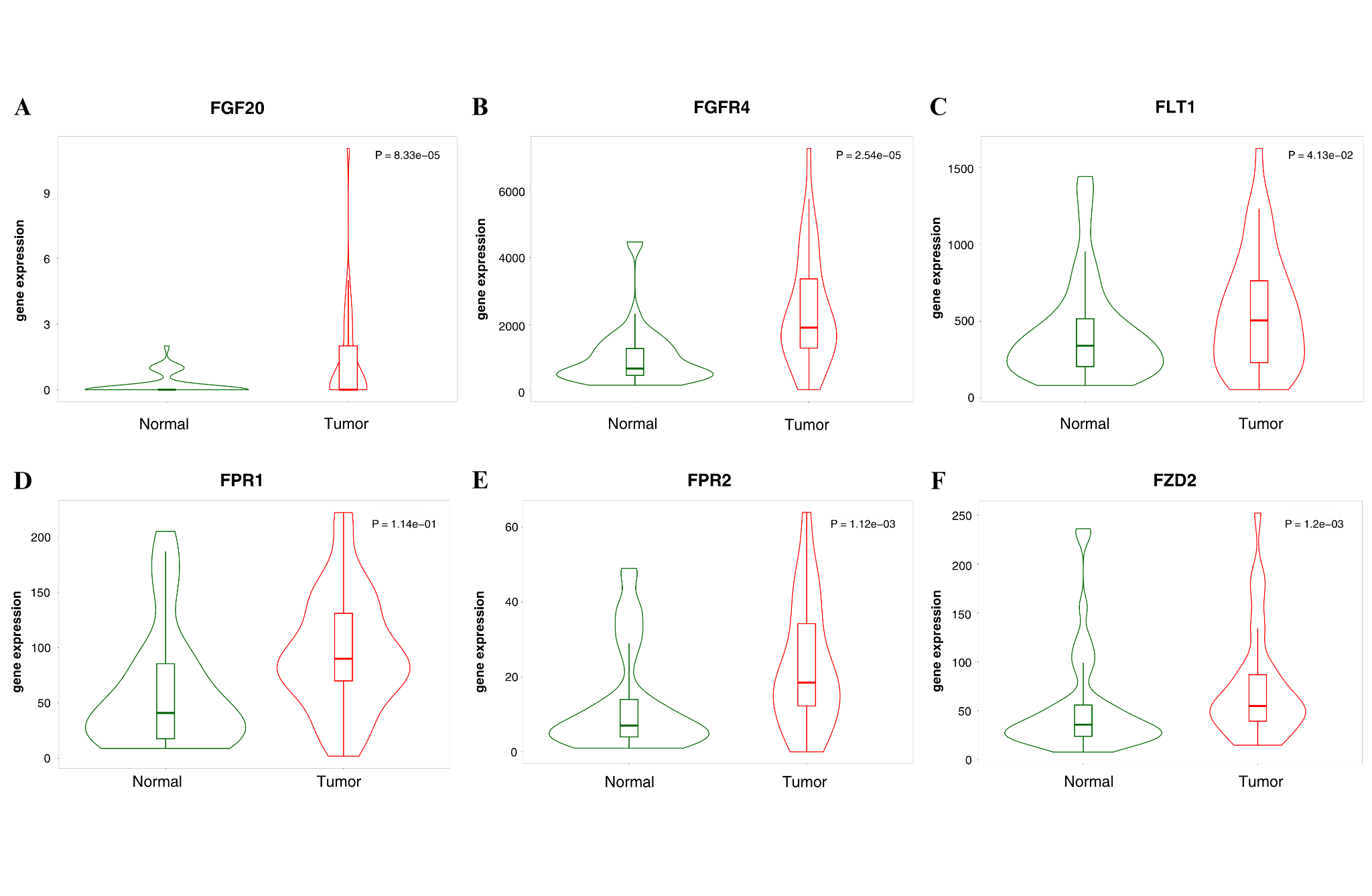

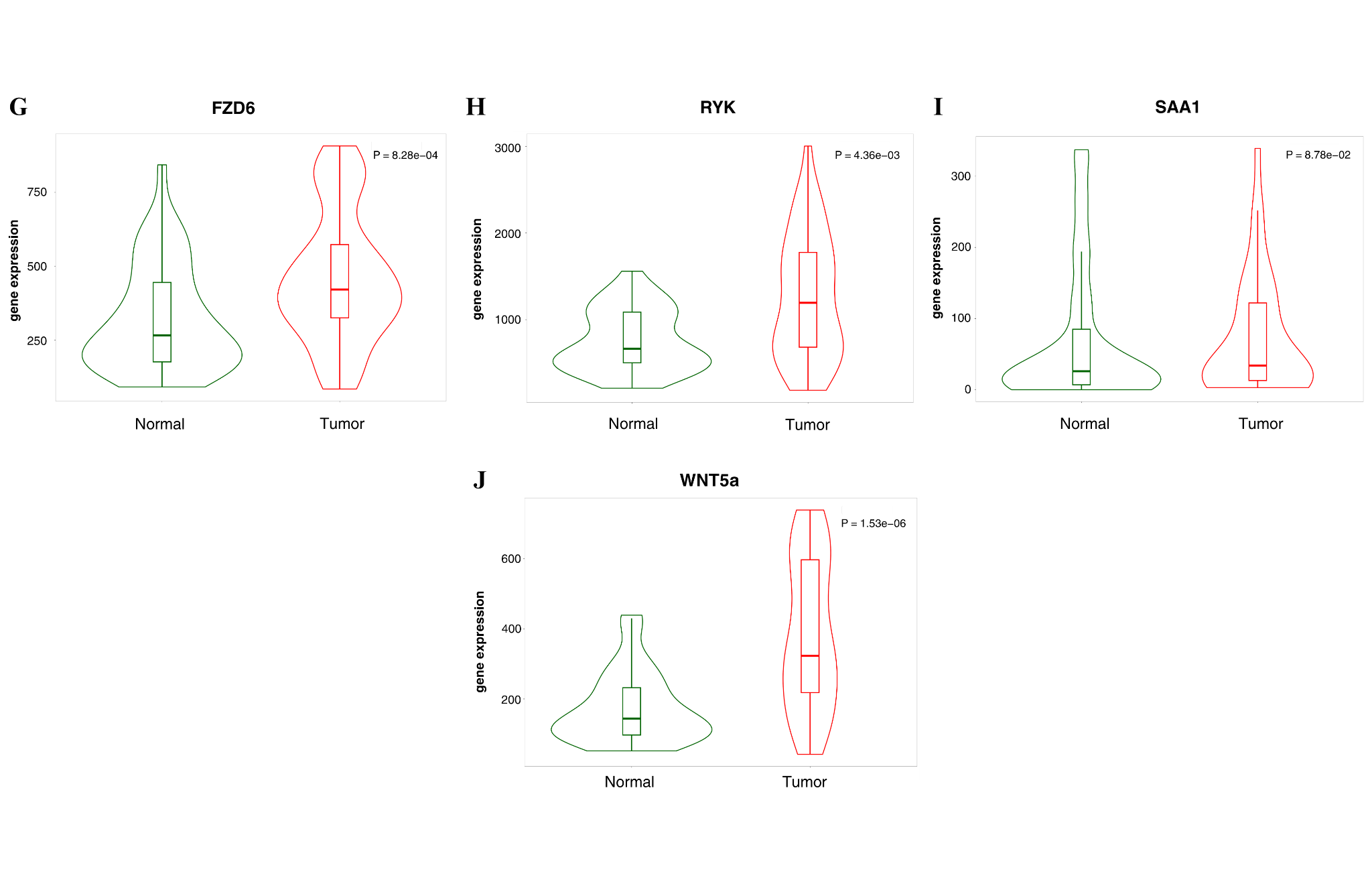


Supplementary fig 4B: Violin plot of the expression of potential biomarkers detected in our analysis in the RNA-Seq of Colon samples database in the TNMplot tool.


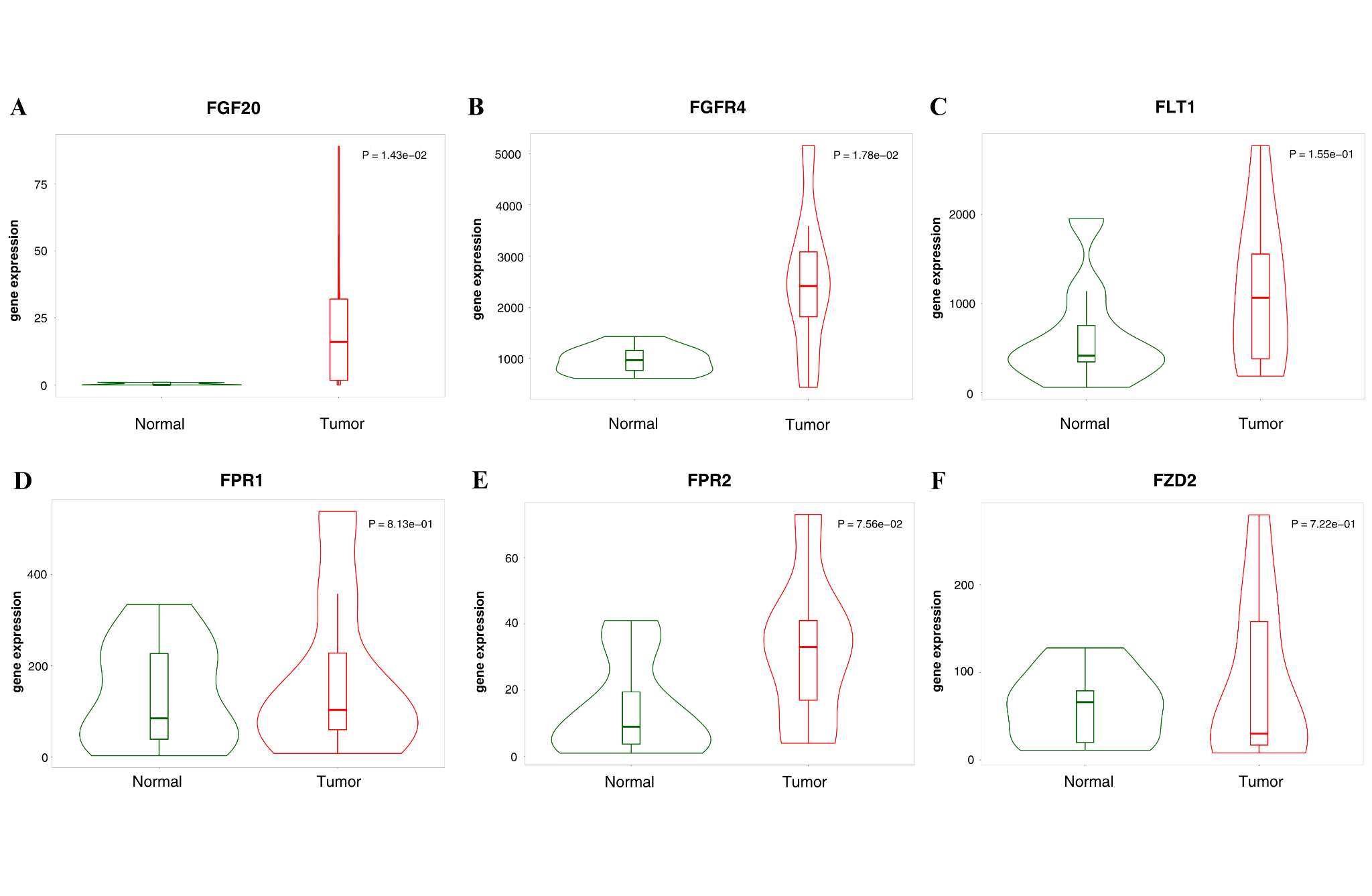

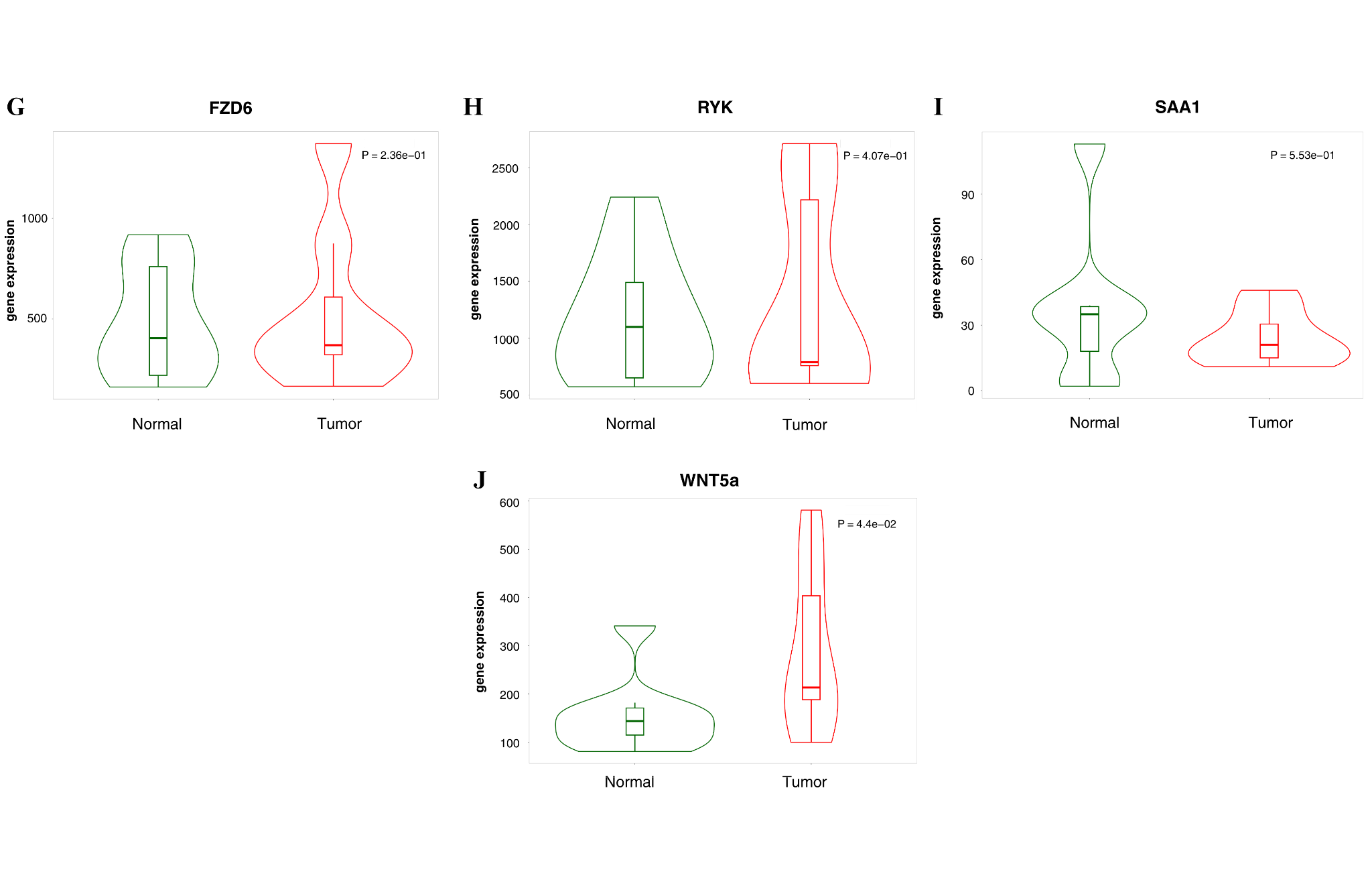


Supplementary fig 4C: Violin plot of the expression of potential biomarkers detected in our analysis in the RNA-seq of Rectum samples database in the TNMplot tool.

Supplementary Fig. 5: Fold change values for the genes detected in the significant pairs across different CRC sites.
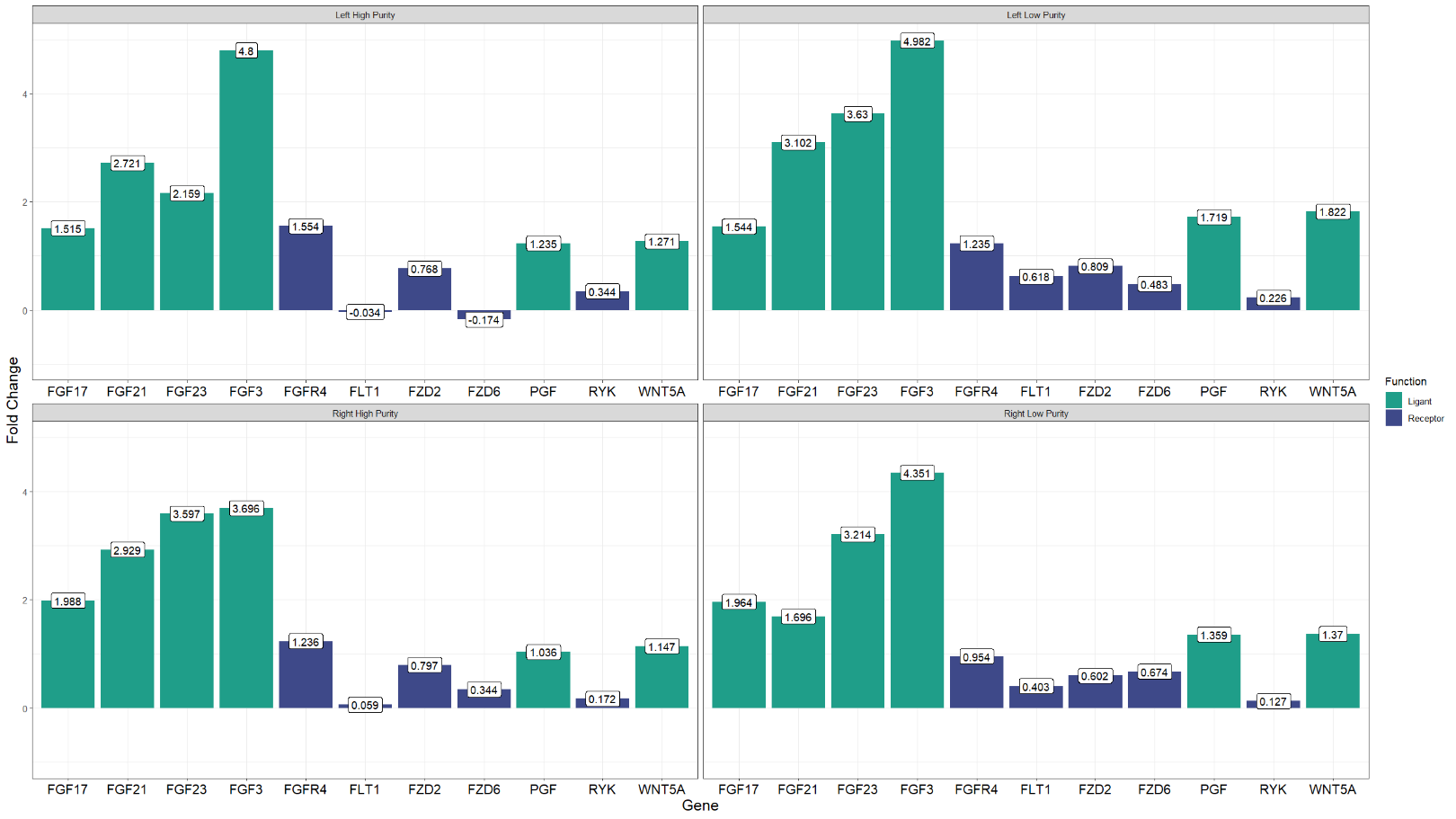


**
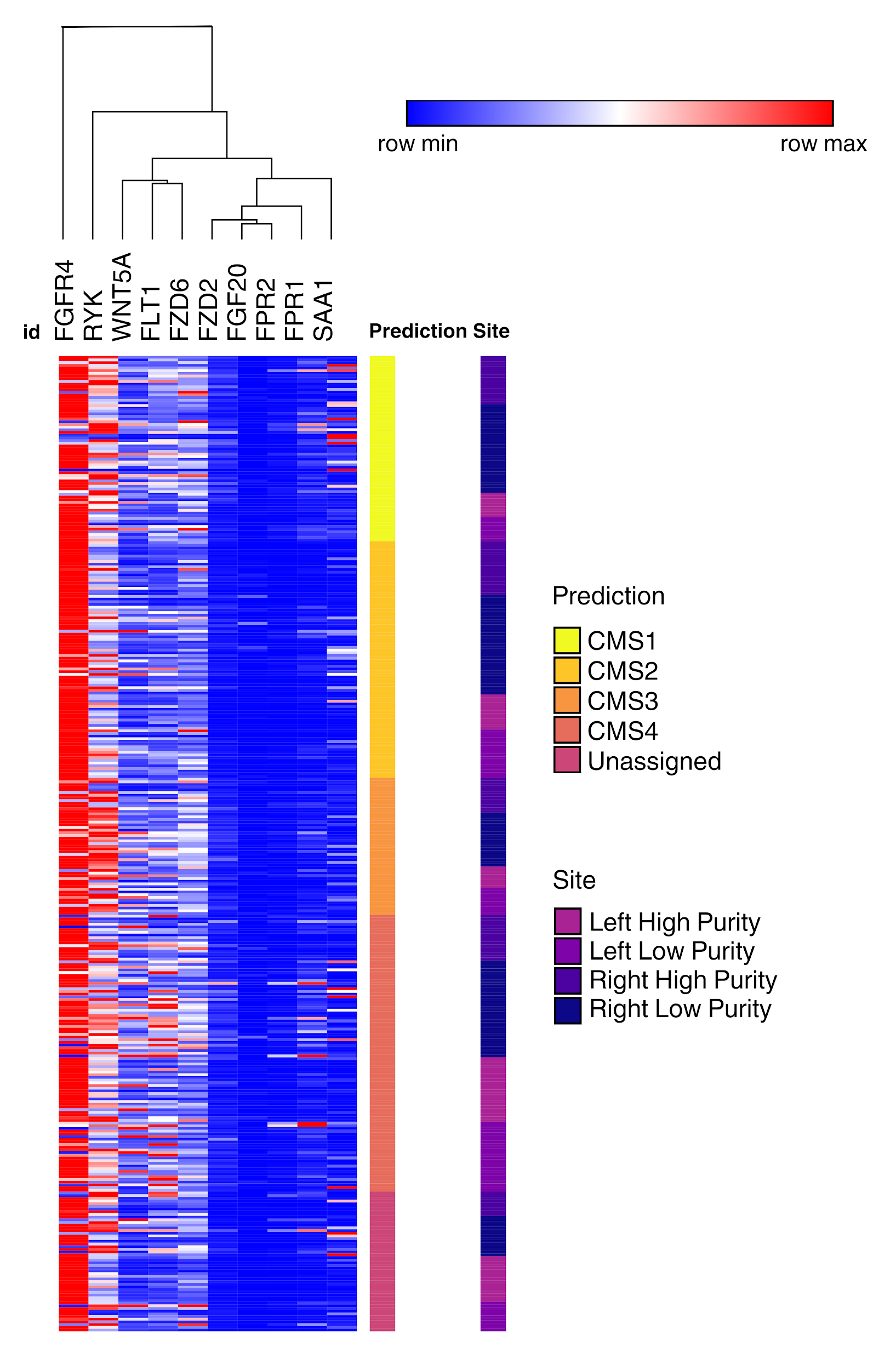
**

Supplementary Fig. 6:Expression of the 10 potential biomarkers detected through interactome analysis according to the CMS identified in different regions. Samples were classified using the CMScaller package, and counts were normalized using Z-score using the Morpheus platform.


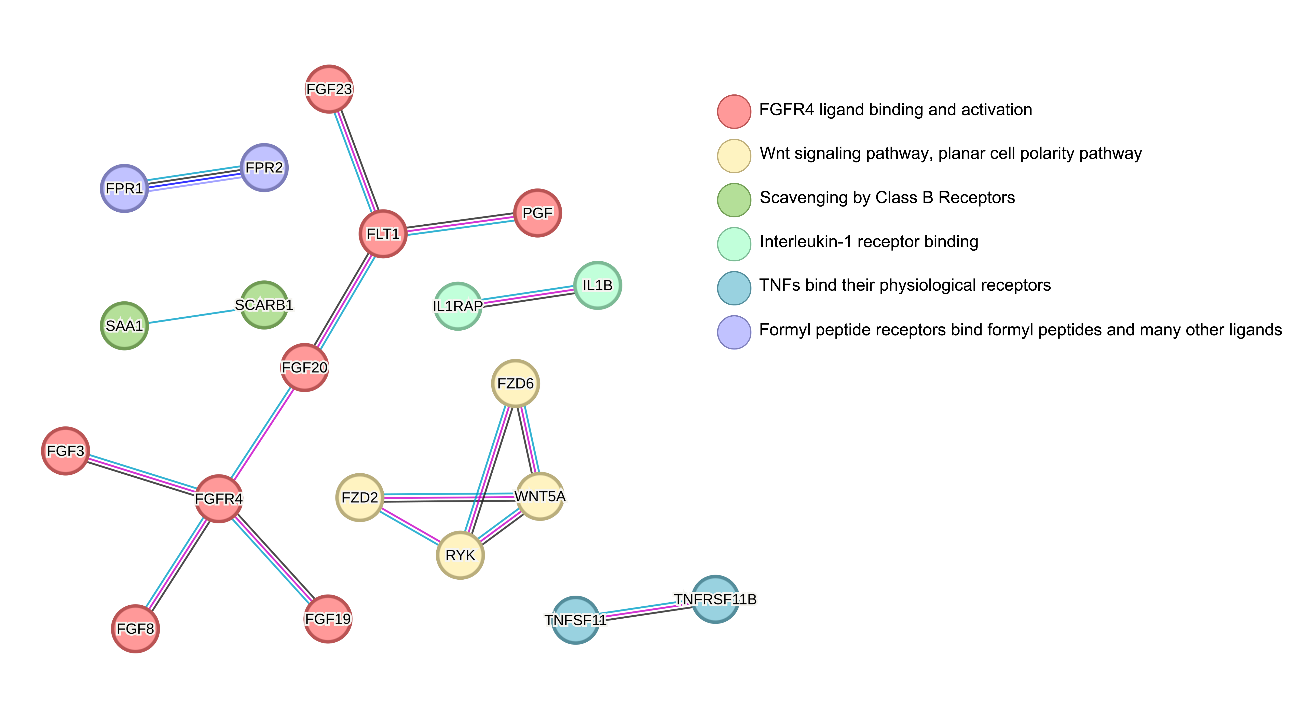


Supplementary Fig. 7: Protein-protein interaction network generated using the STRING tool, enriched and clustered by k-means. Six clusters related to distinct biological processes were identified: Red: FGFR4 ligand binding and activation. Yellow: Wnt signaling pathway, planar cell polarity pathway. Lime Green: Elimination by class B receptors. Green: Interleukin-1 receptor binding. Blue: TNF binding its physiological receptors. Purple: Formyl peptide receptors binding formyl peptides and various other ligands.

**
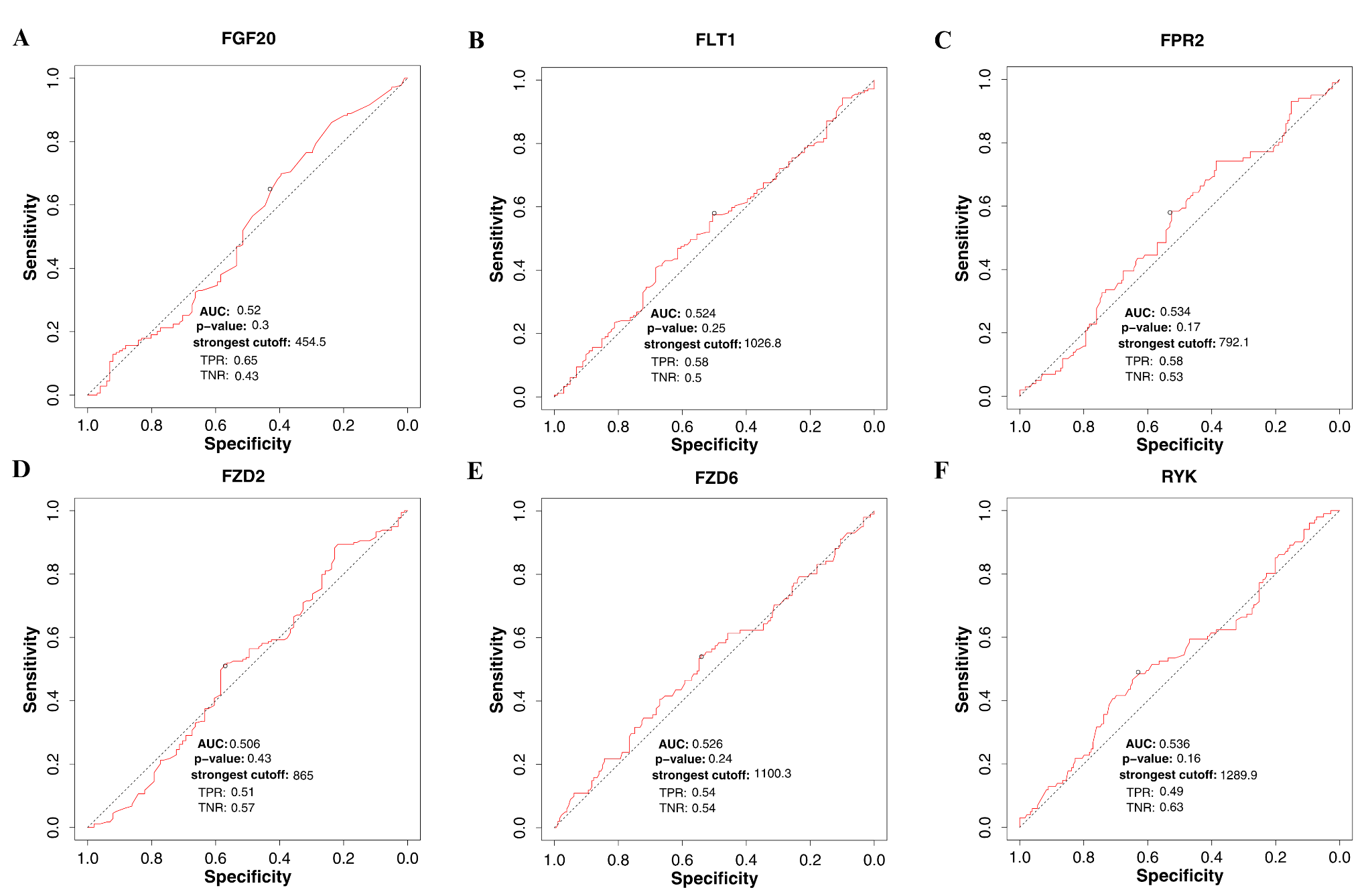
**

**
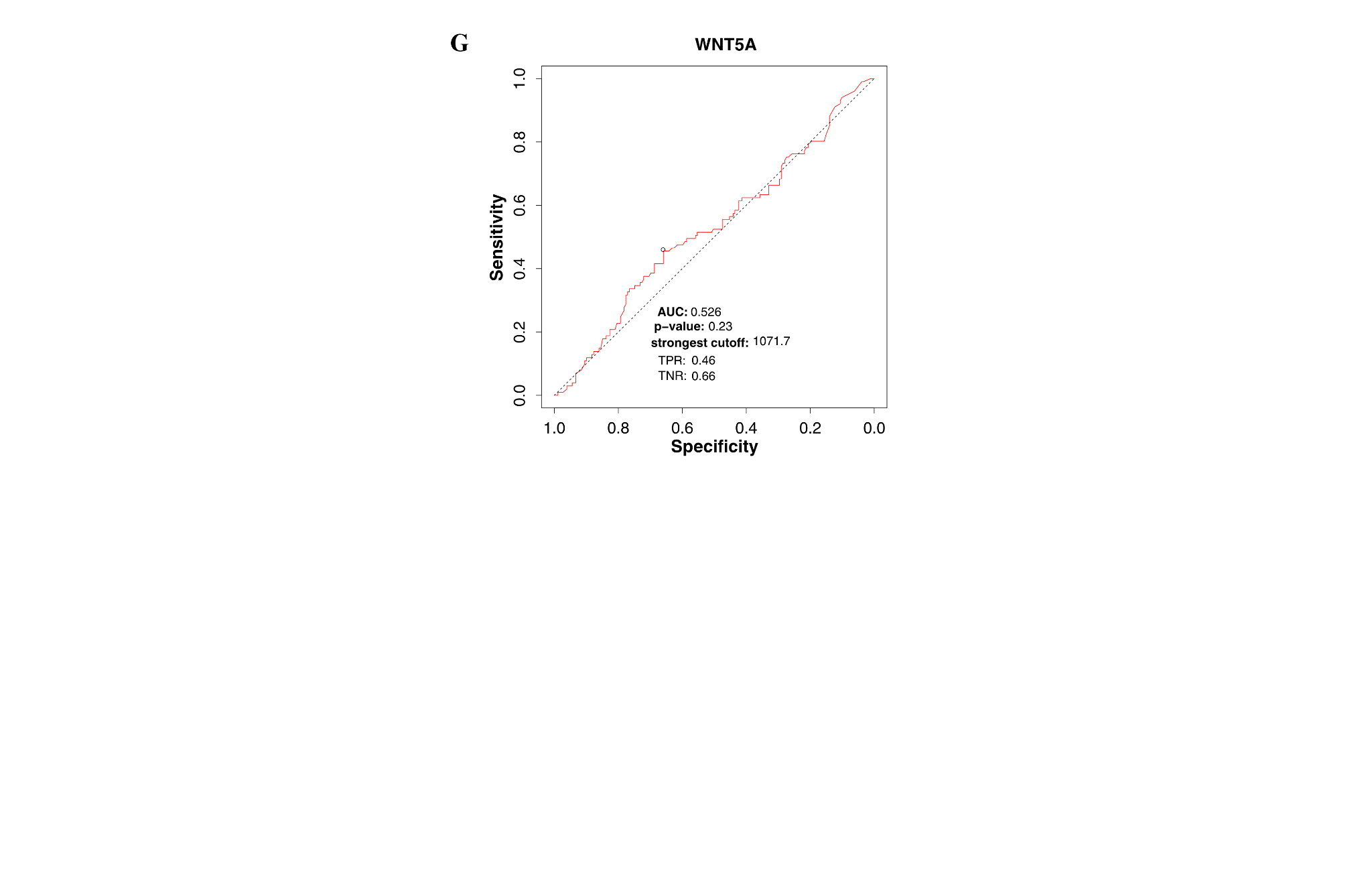
**

Supplementary Fig. 8: ROC curves for the markers identified in our analysis for the colon, using the TNMplot tool. The genes did not show significant values as good therapeutic predictors for this region.


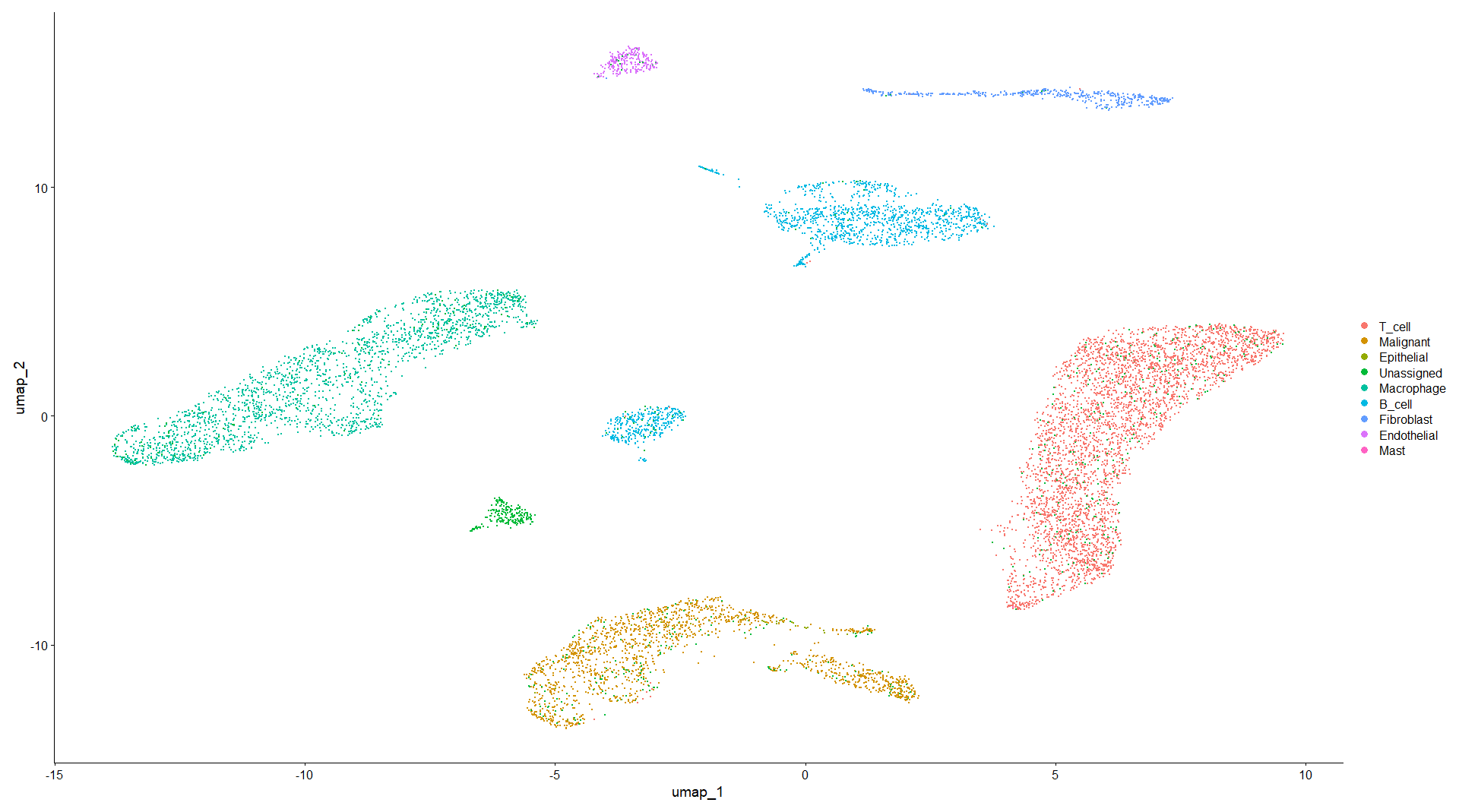


Supplementary Fig. 9: UMAP plot displaying the cell types identified from the data of Lee et al., 2020. Eight cell types were identified in the tumor microenvironment of samples extracted from the large intestine: T cells, malignant cells, epithelial cells, macrophages, B cells, fibroblasts, endothelial cells, and mast cells.


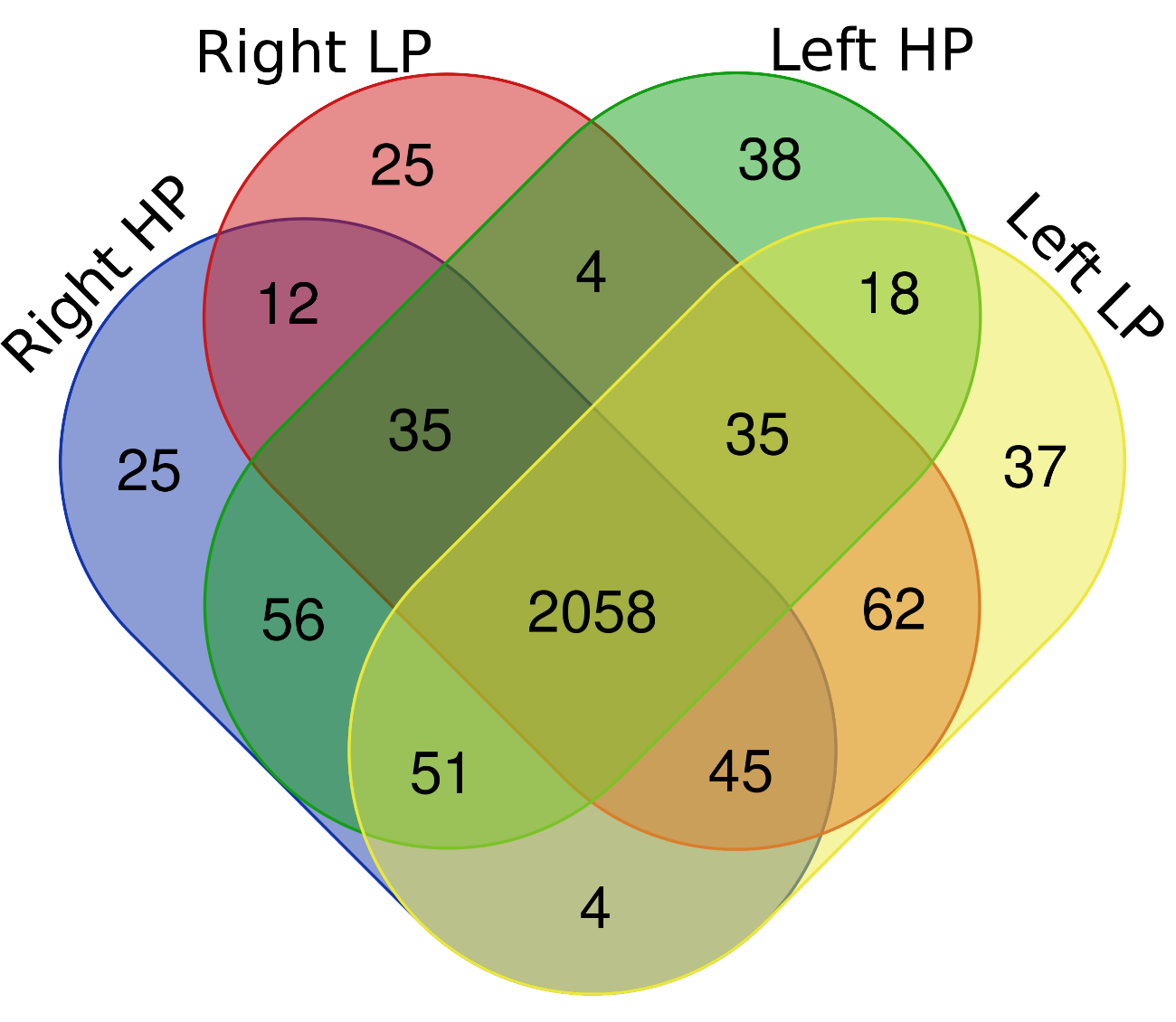


Supplementary Fig. 10: Venn diagram illustrating the drugs predicted to reverse the expression profile, based on the connectivity map obtained from the L1000FWD platform.


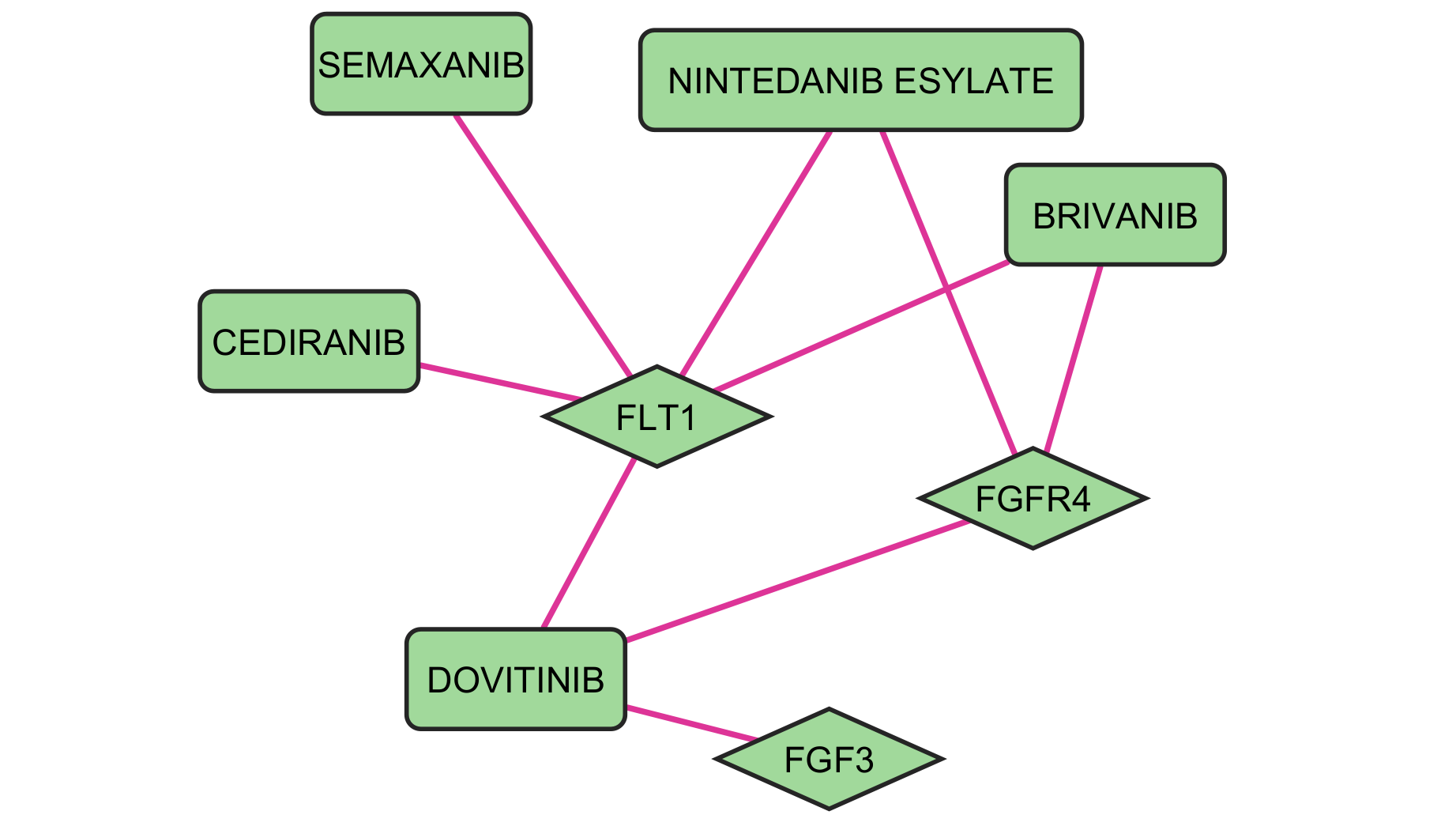


Supplementary Fig. 11: Interaction network of drugs identified in more than one tool and their respective targets predicted by the DGIDB tool.
