# Supplementary figures and images for: Integrating the secretome and interactome to identify novel biomarkers and therapeutic targets in colorectal cancer
Source: Cell Commun Signal. 2025 Oct 10;23:428. doi: 10.1186/s12964-025-02424-4 (PMC12512465; doi:10.1186/s12964-025-02424-4)

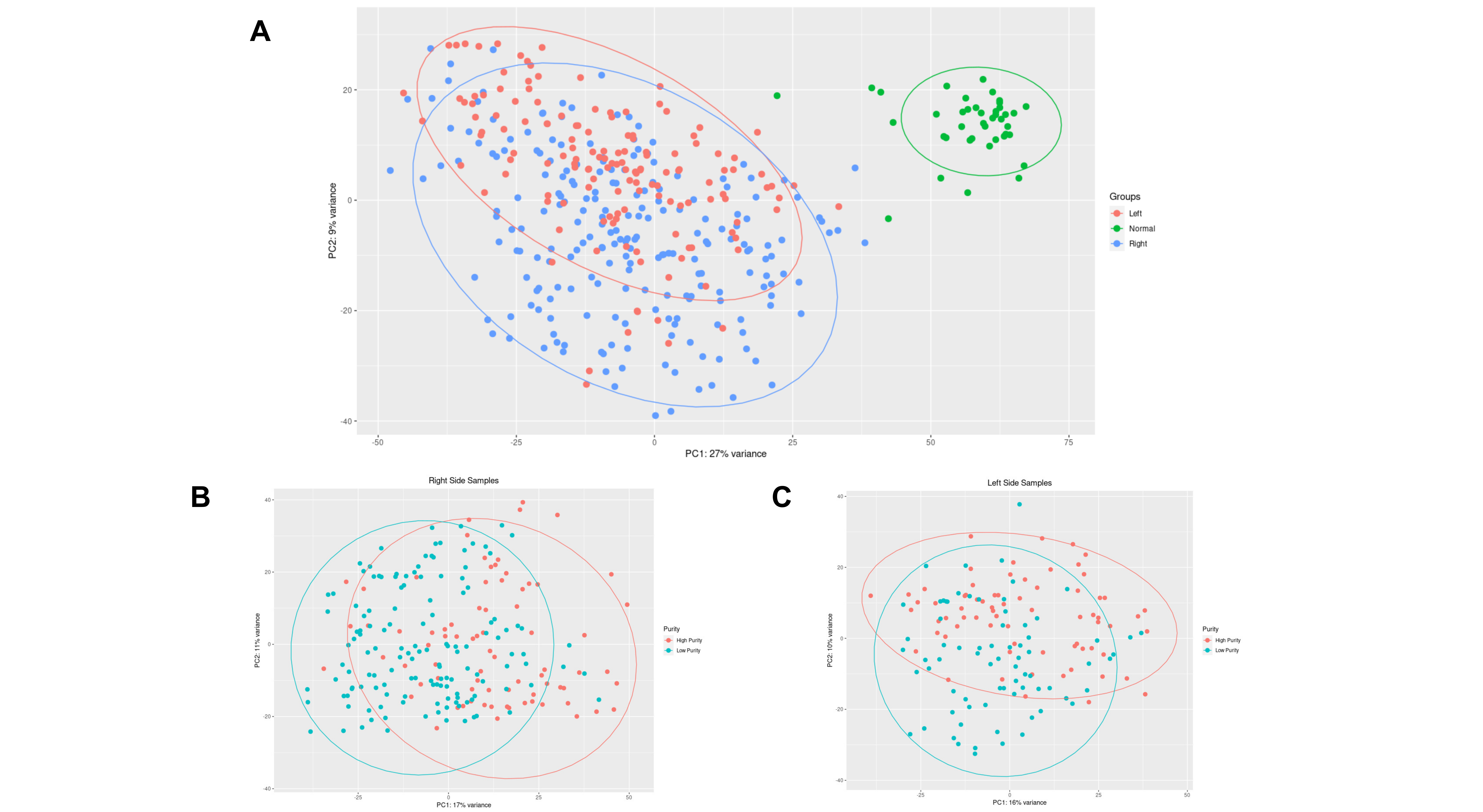

Supplement: Supplementary file 1 — Supplementary Material 1. [file 12964_2025_2424_MOESM1_ESM.zip › Supplementary/Sup 1.png]

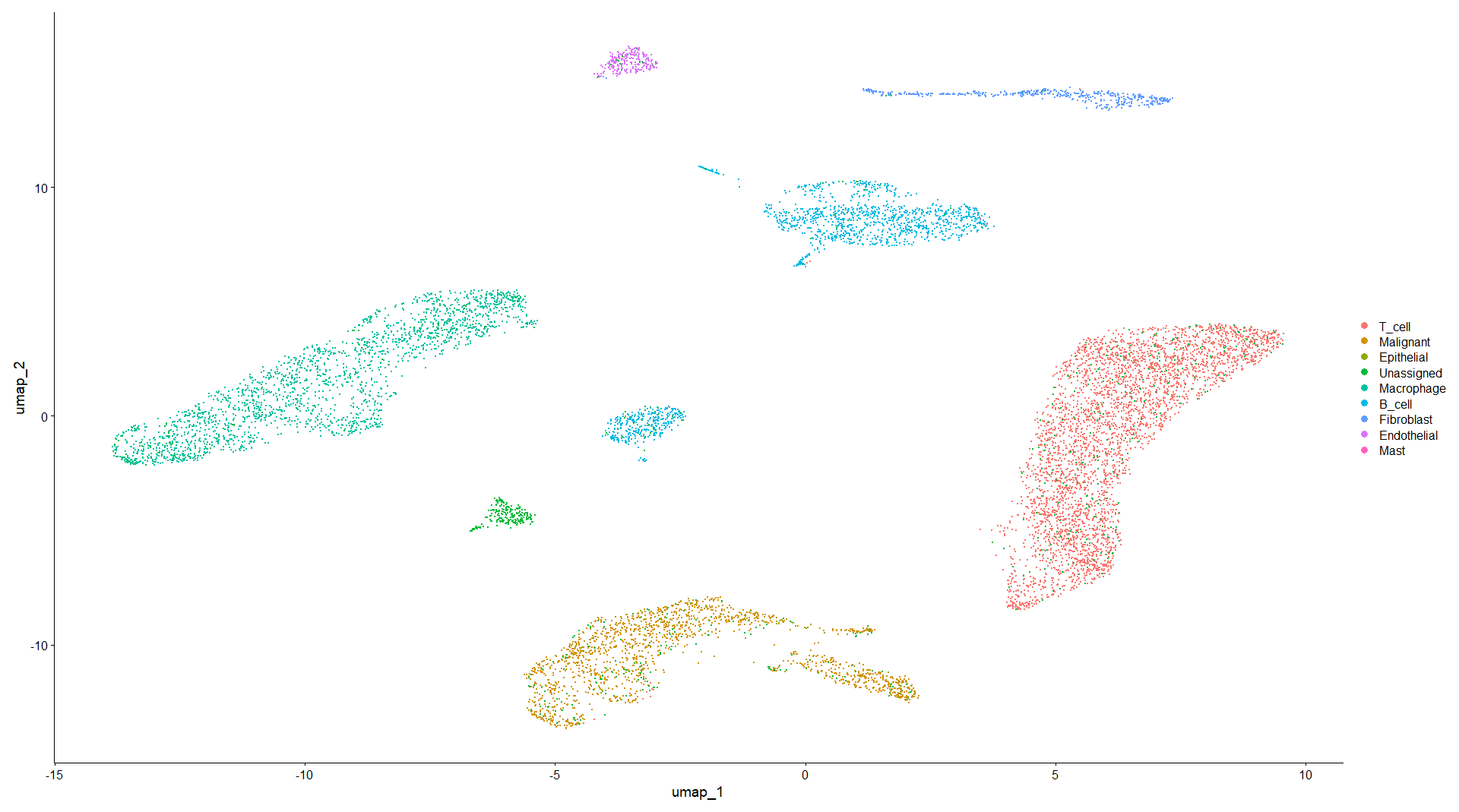

Supplement: Supplementary file 1 — Supplementary Material 1. [file 12964_2025_2424_MOESM1_ESM.zip › Supplementary/Sup 10.png]

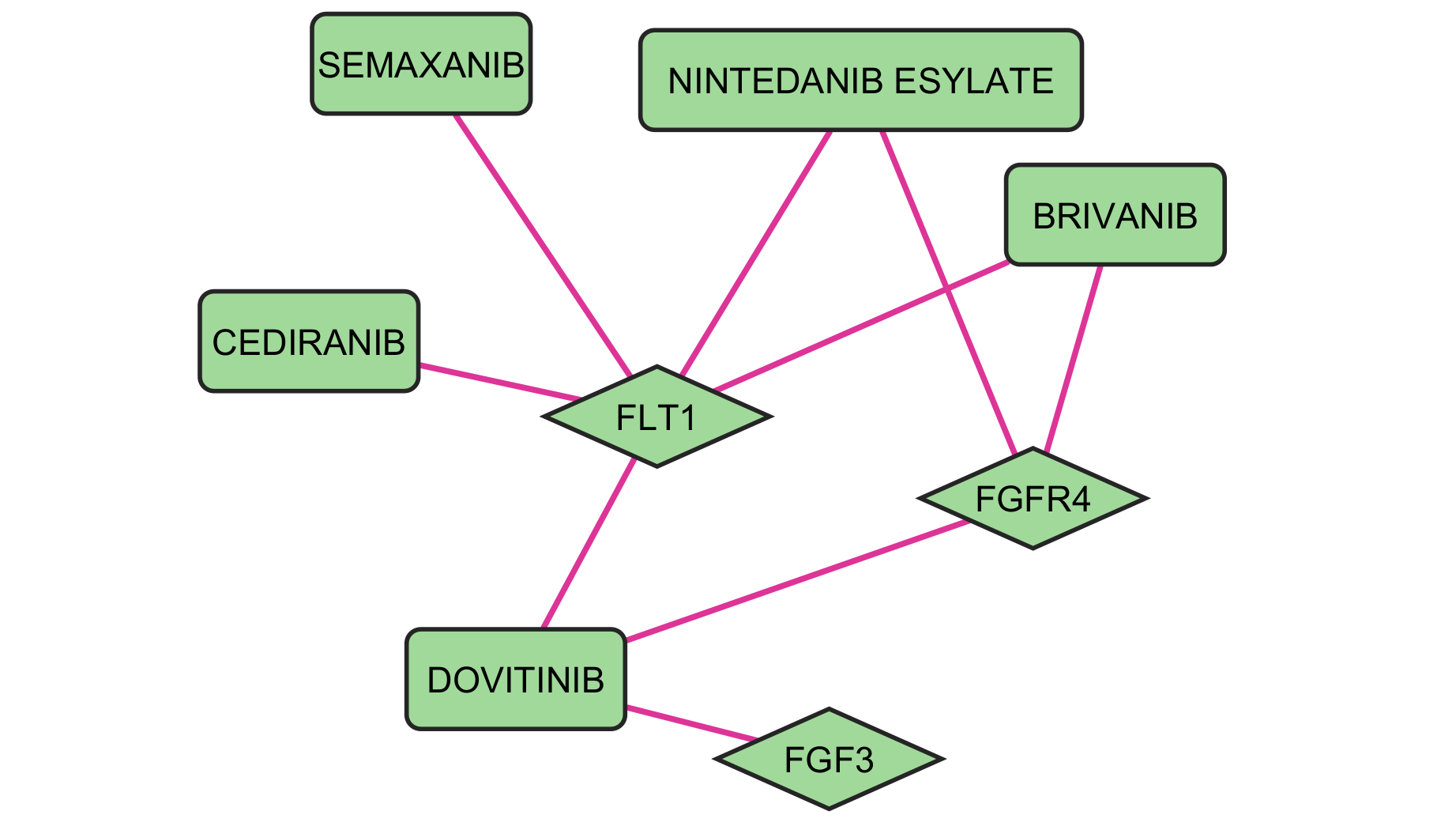

Supplement: Supplementary file 1 — Supplementary Material 1. [file 12964_2025_2424_MOESM1_ESM.zip › Supplementary/Sup 11.png]

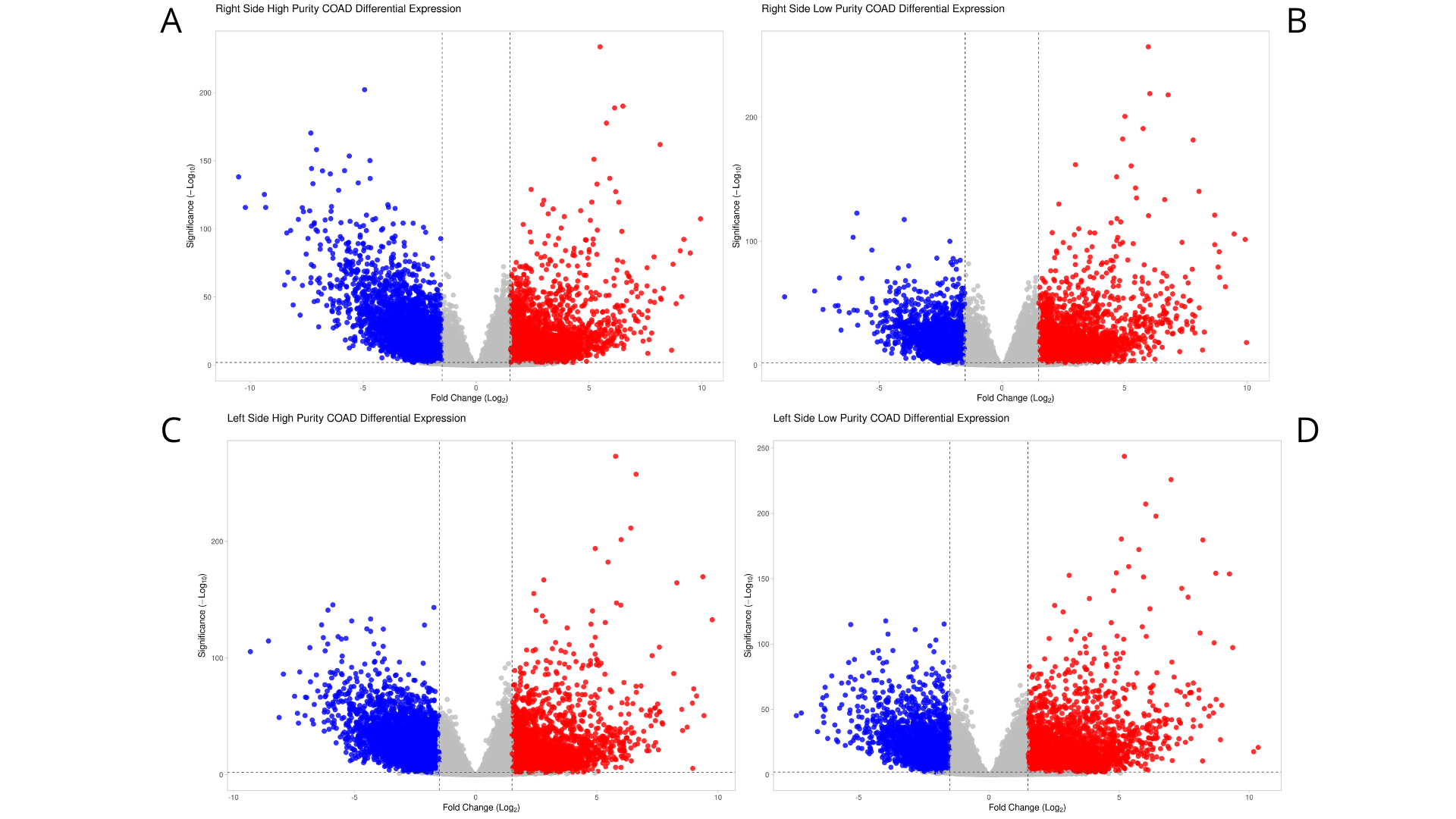

Supplement: Supplementary file 1 — Supplementary Material 1. [file 12964_2025_2424_MOESM1_ESM.zip › Supplementary/Sup 2.png]

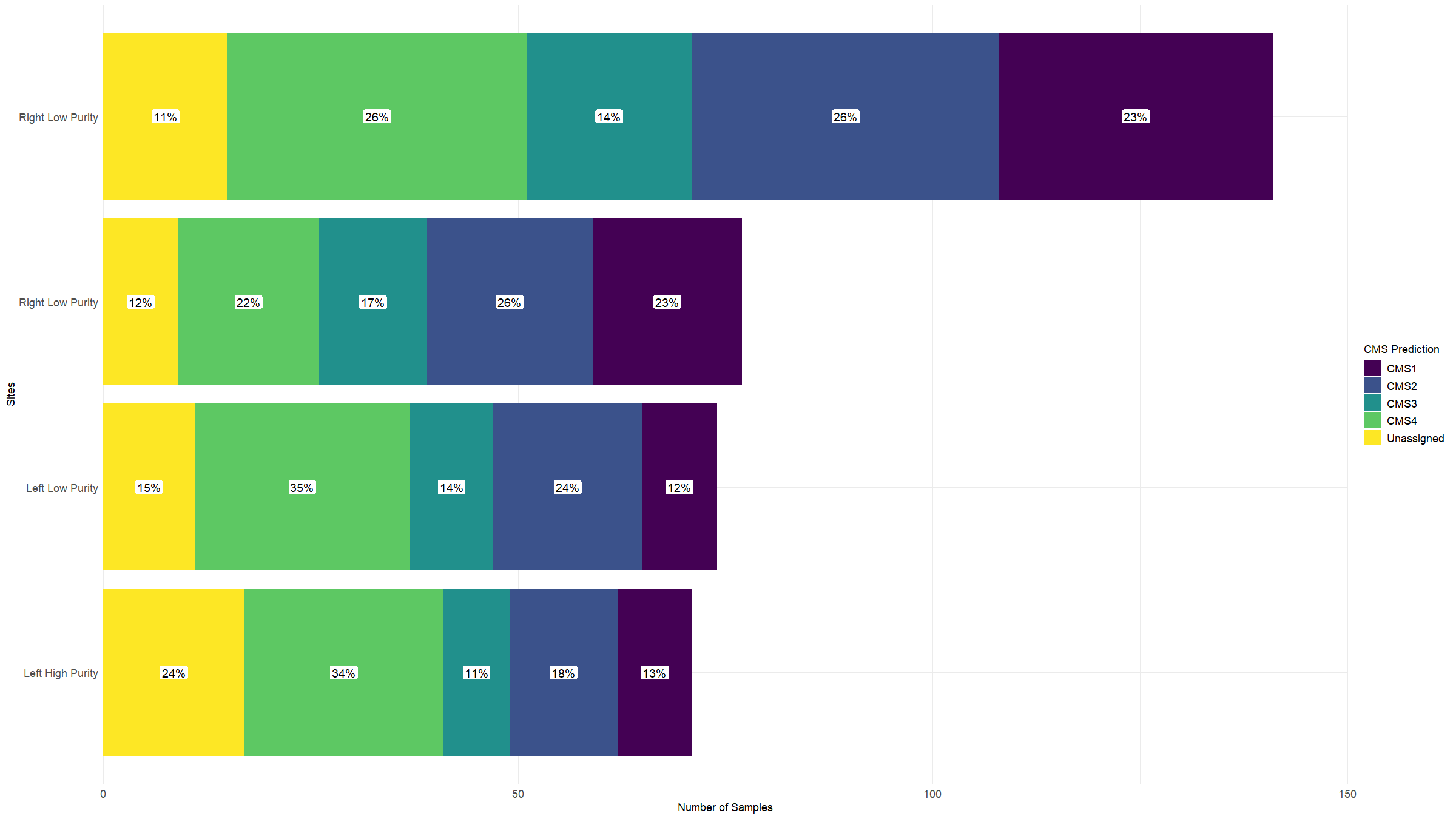

Supplement: Supplementary file 1 — Supplementary Material 1. [file 12964_2025_2424_MOESM1_ESM.zip › Supplementary/Sup 3.png]

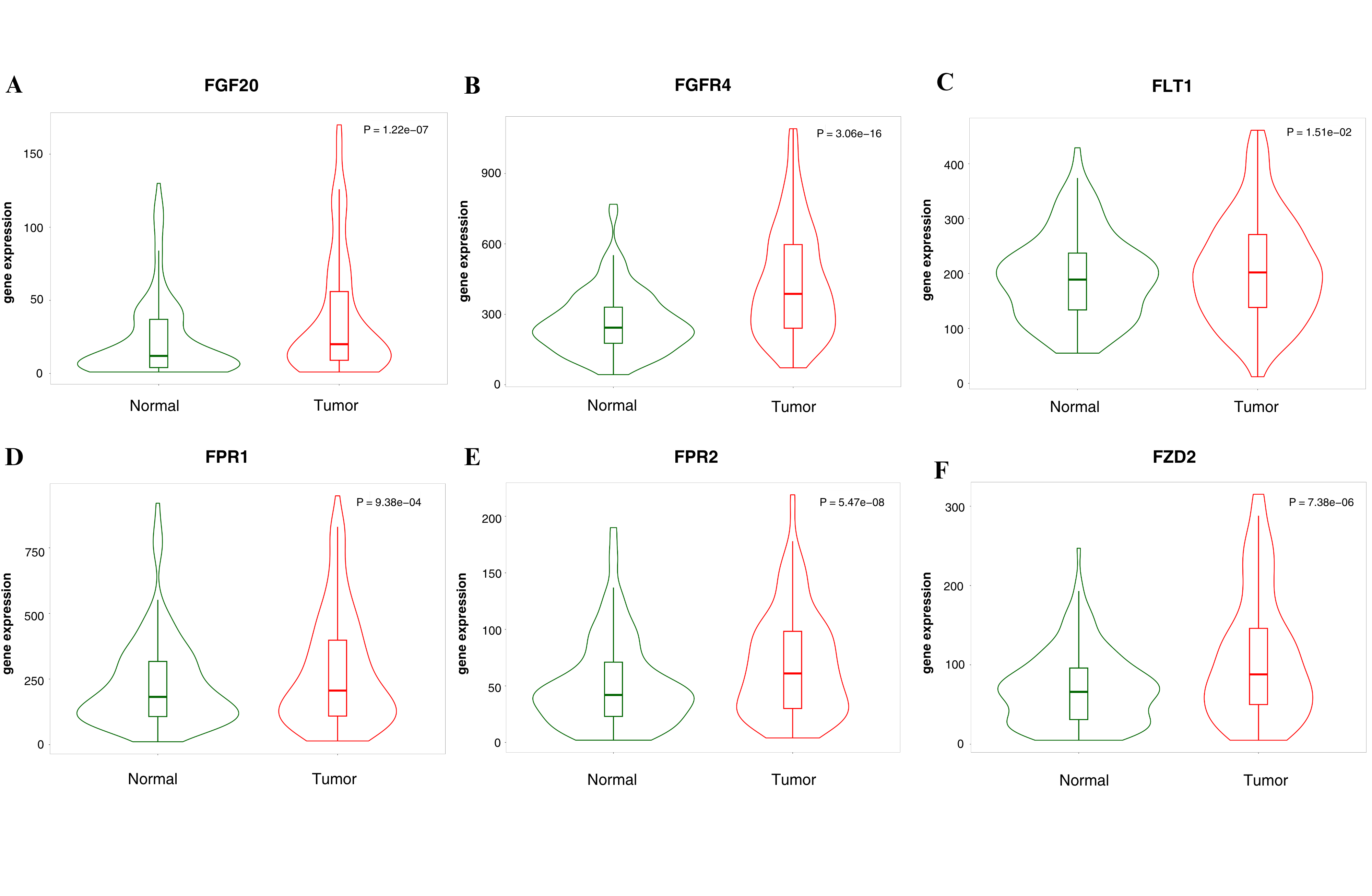

Supplement: Supplementary file 1 — Supplementary Material 1. [file 12964_2025_2424_MOESM1_ESM.zip › Supplementary/Sup 4a.1.png]

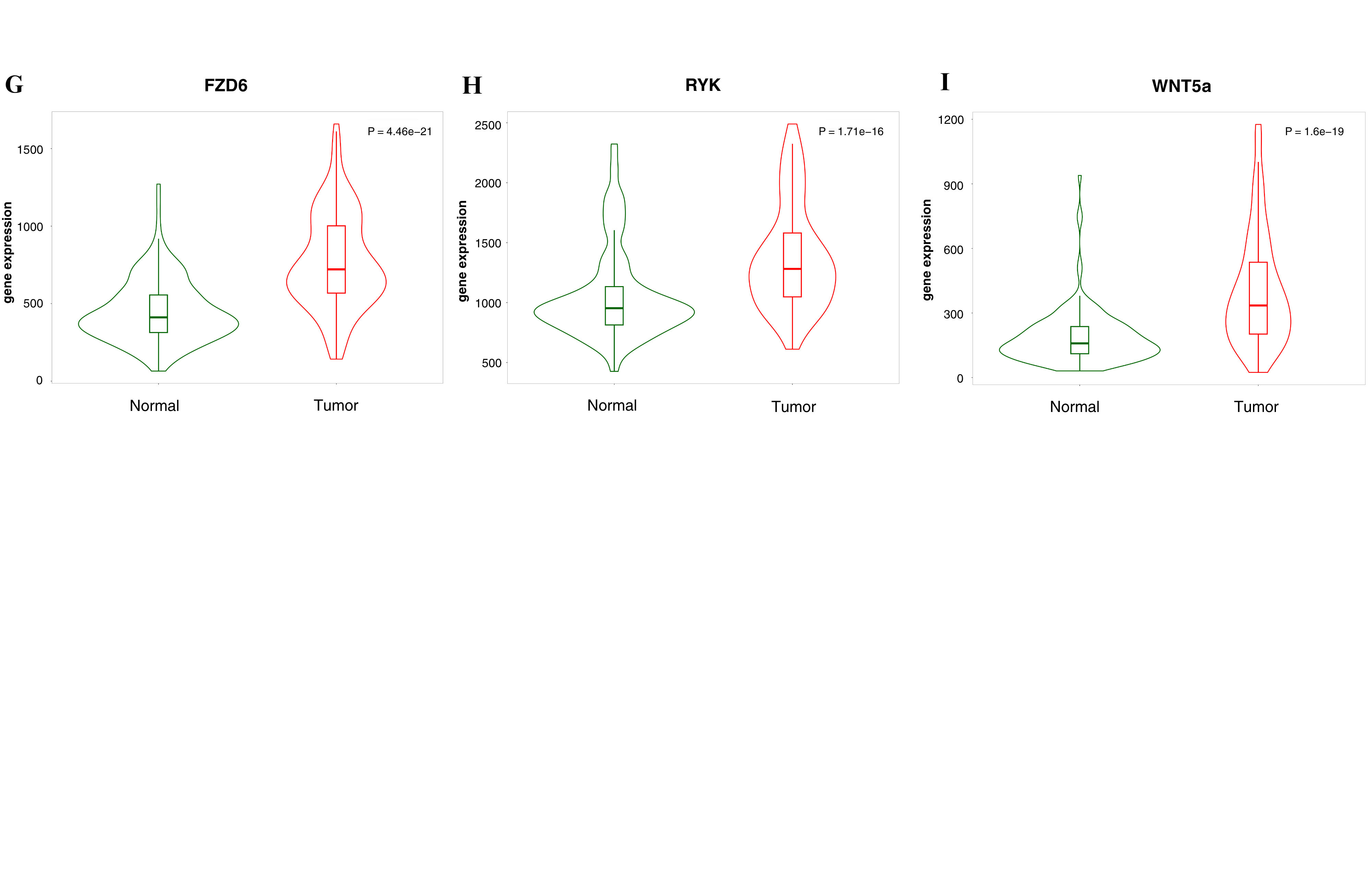

Supplement: Supplementary file 1 — Supplementary Material 1. [file 12964_2025_2424_MOESM1_ESM.zip › Supplementary/Sup 4a.2.png]

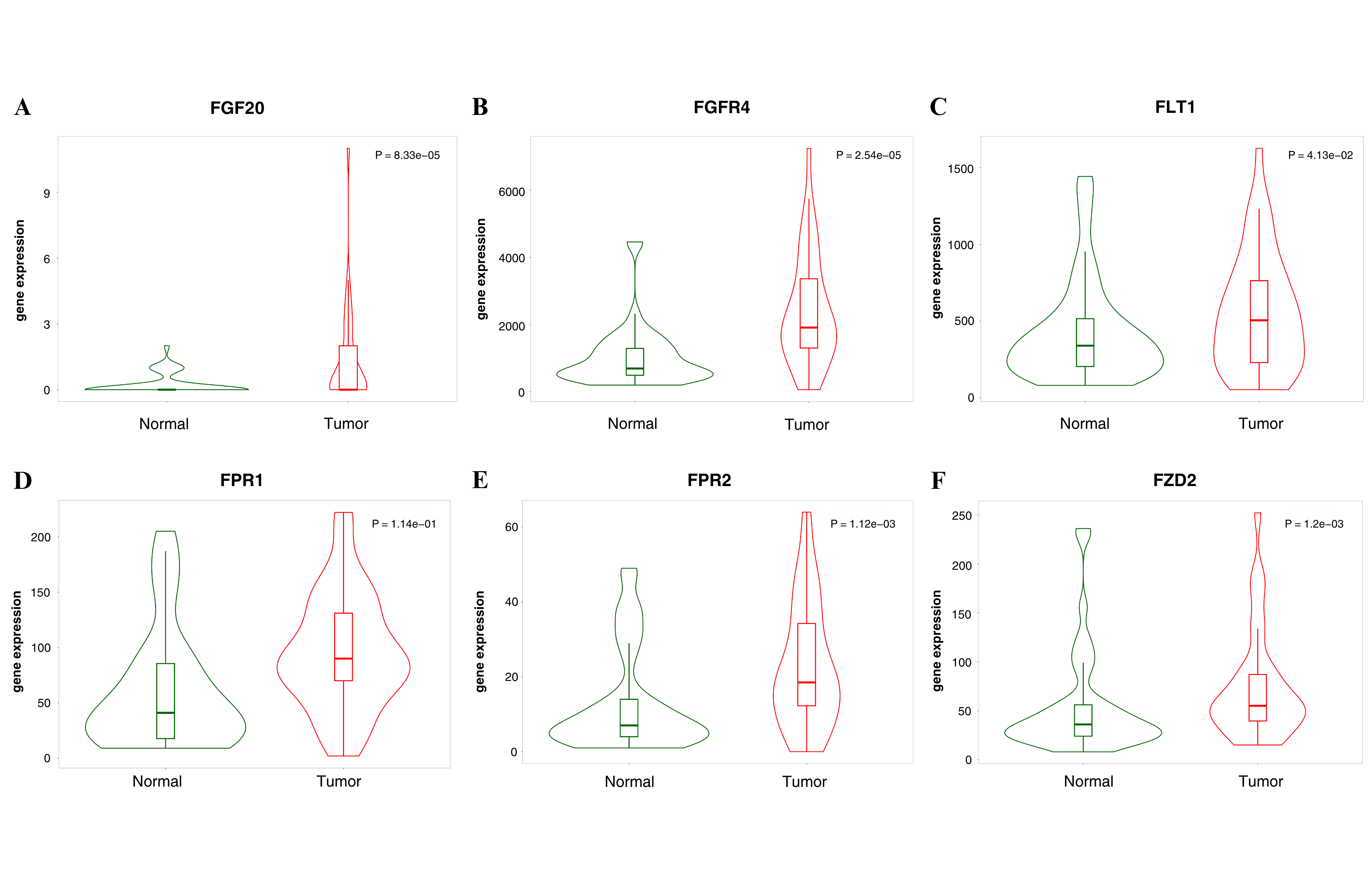

Supplement: Supplementary file 1 — Supplementary Material 1. [file 12964_2025_2424_MOESM1_ESM.zip › Supplementary/Sup 4b.1.png]

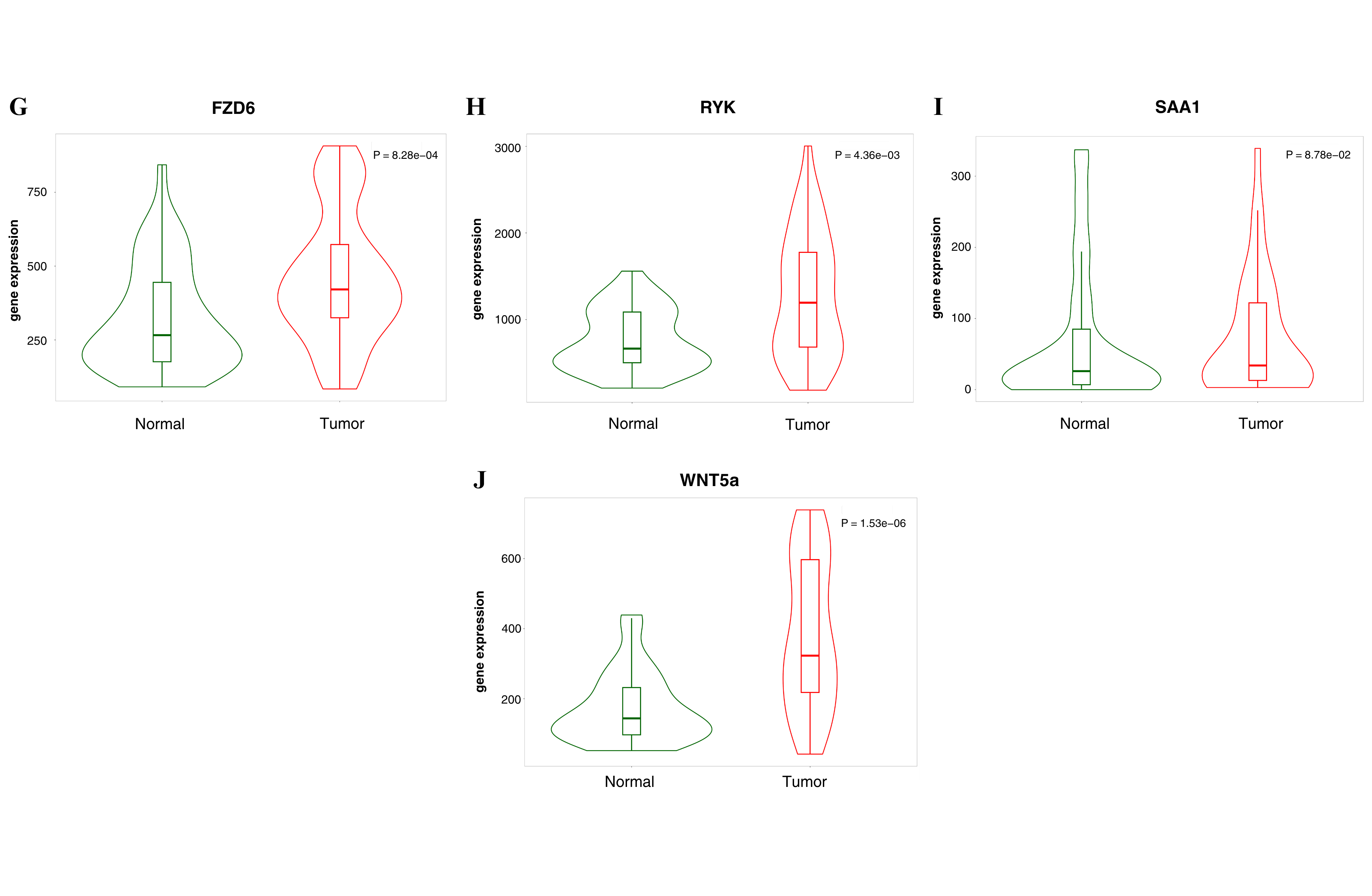

Supplement: Supplementary file 1 — Supplementary Material 1. [file 12964_2025_2424_MOESM1_ESM.zip › Supplementary/Sup 4b.2.png]

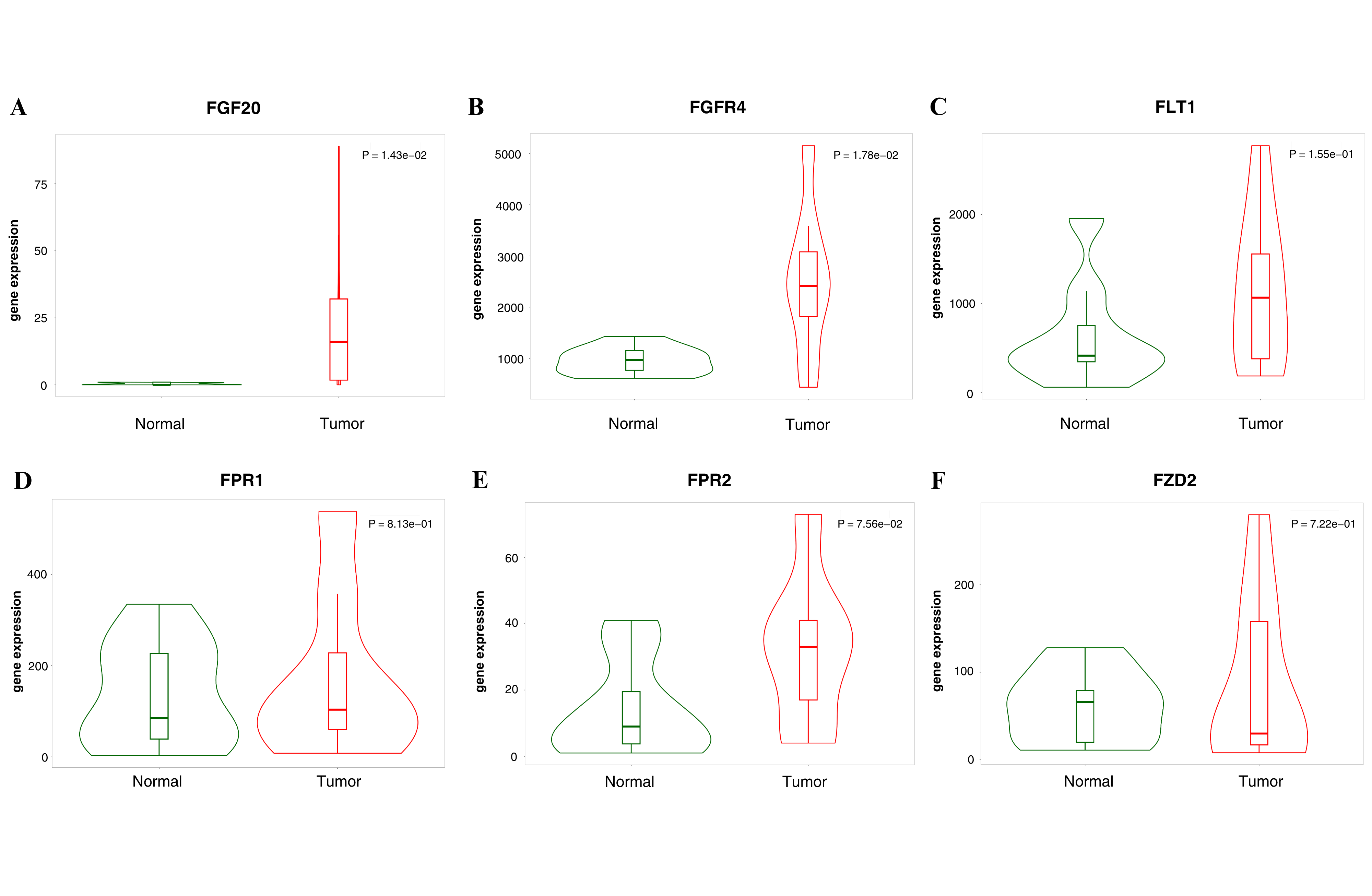

Supplement: Supplementary file 1 — Supplementary Material 1. [file 12964_2025_2424_MOESM1_ESM.zip › Supplementary/Sup 4c.1.png]

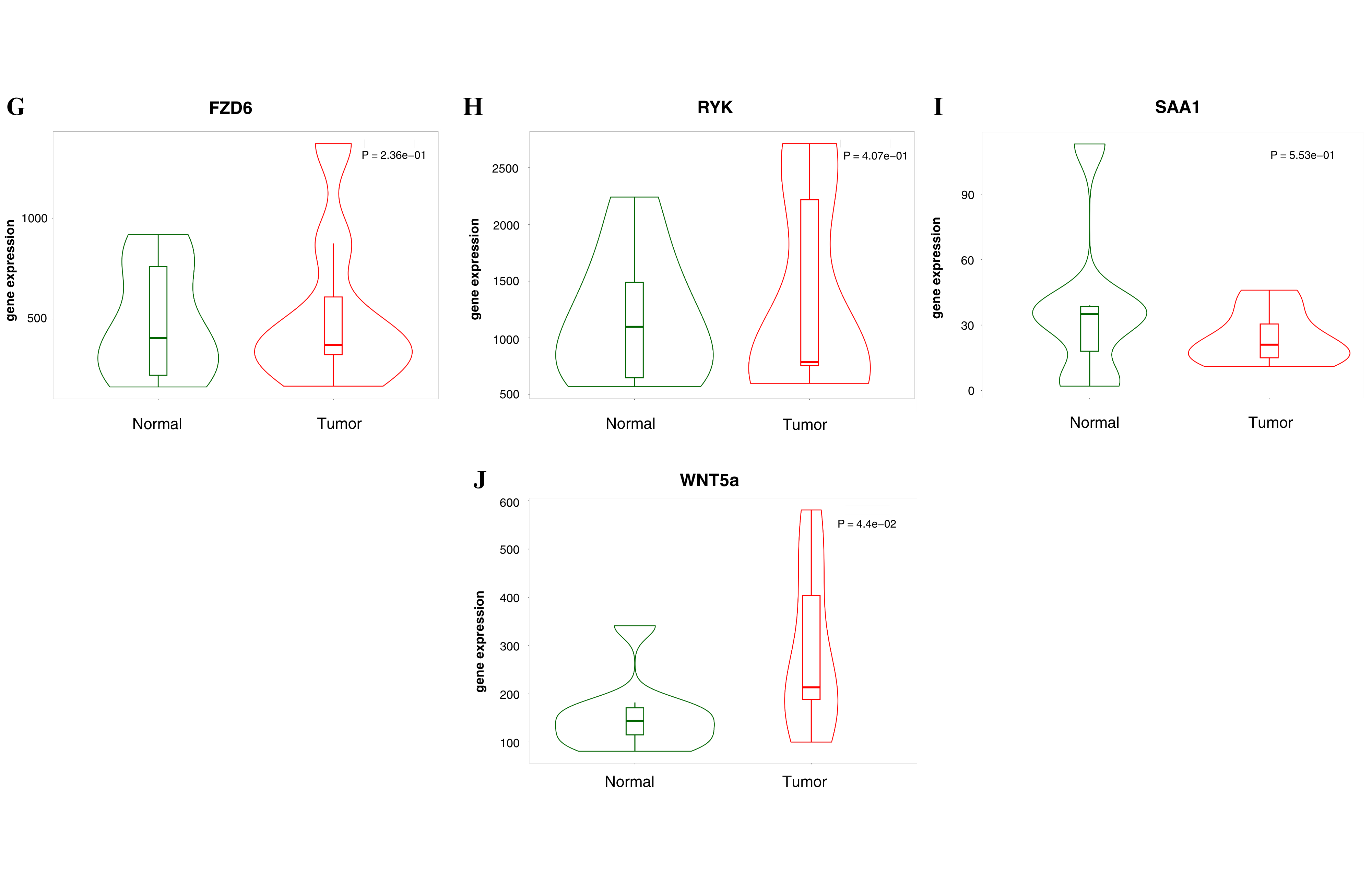

Supplement: Supplementary file 1 — Supplementary Material 1. [file 12964_2025_2424_MOESM1_ESM.zip › Supplementary/Sup 4c.2.png]

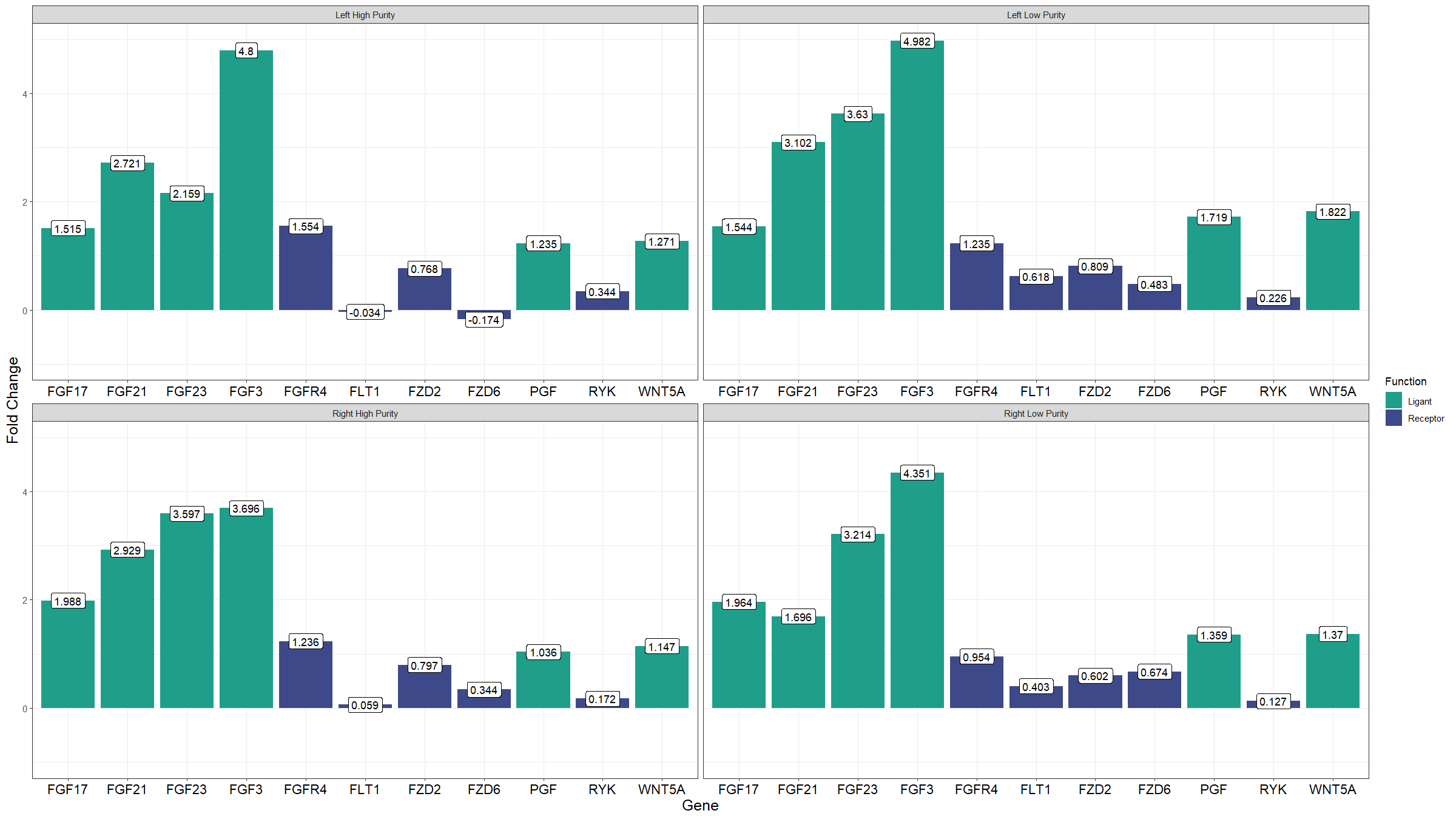

Supplement: Supplementary file 1 — Supplementary Material 1. [file 12964_2025_2424_MOESM1_ESM.zip › Supplementary/Sup 5.tiff]

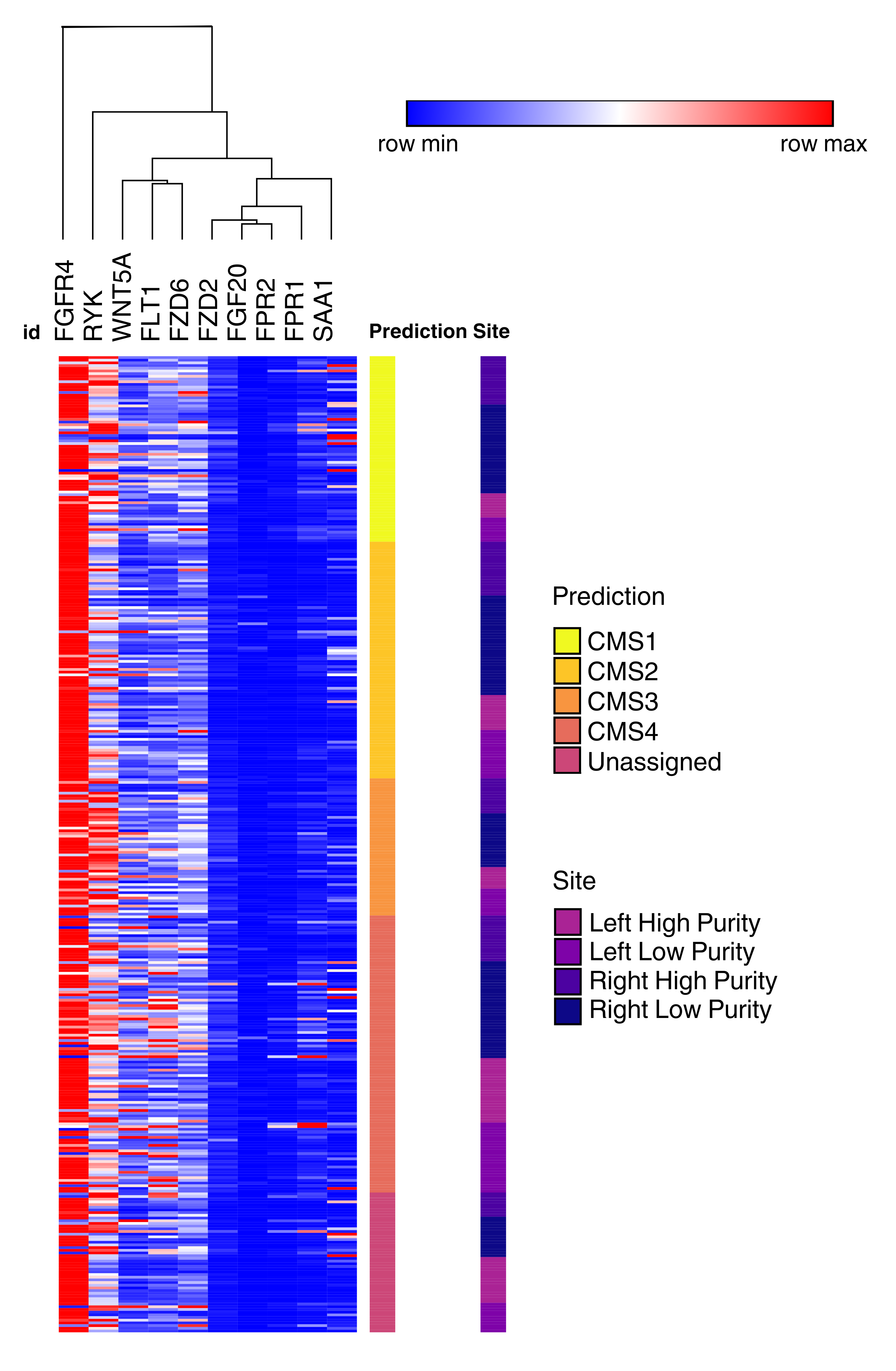

Supplement: Supplementary file 1 — Supplementary Material 1. [file 12964_2025_2424_MOESM1_ESM.zip › Supplementary/Sup 6.png]

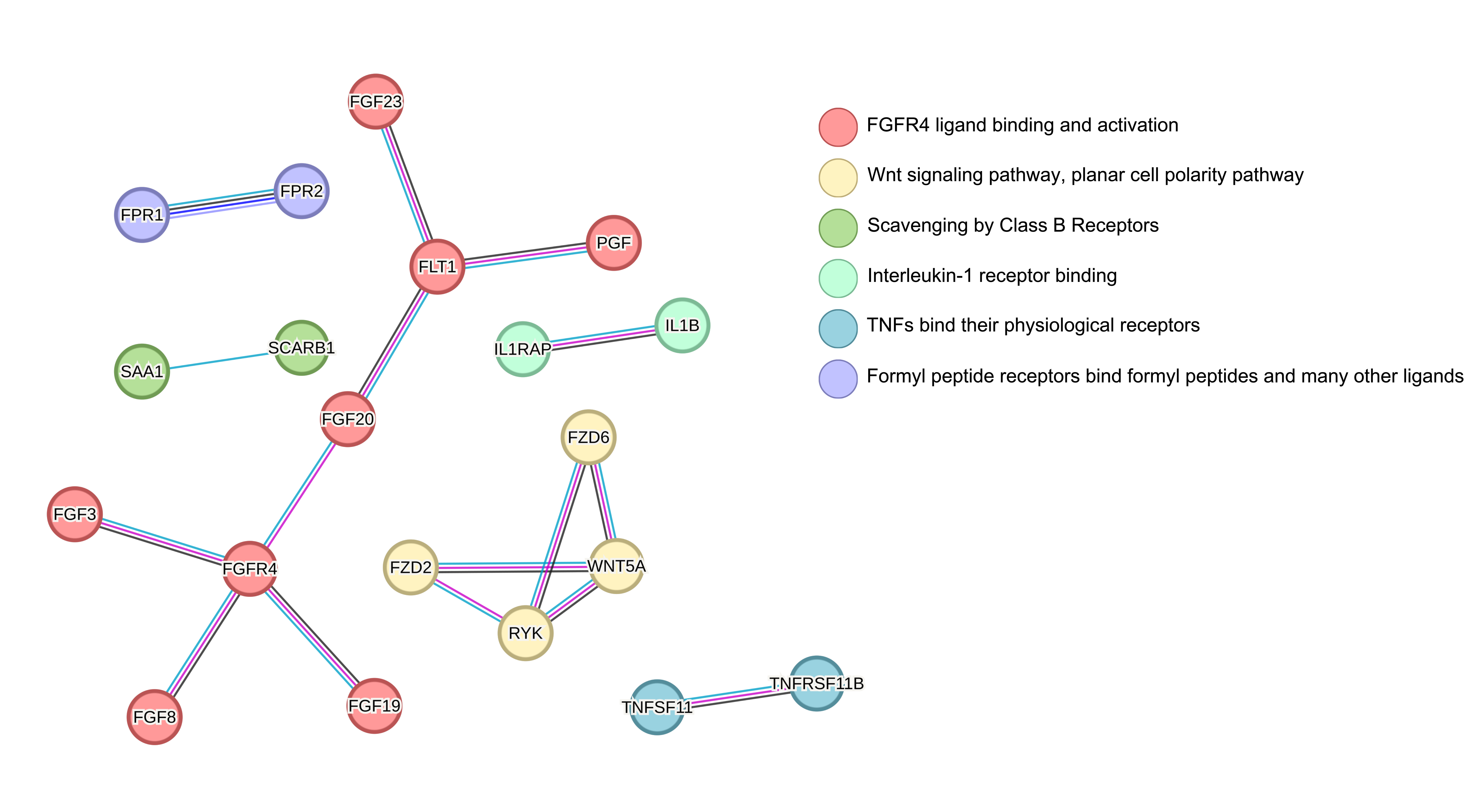

Supplement: Supplementary file 1 — Supplementary Material 1. [file 12964_2025_2424_MOESM1_ESM.zip › Supplementary/Sup 7.png]

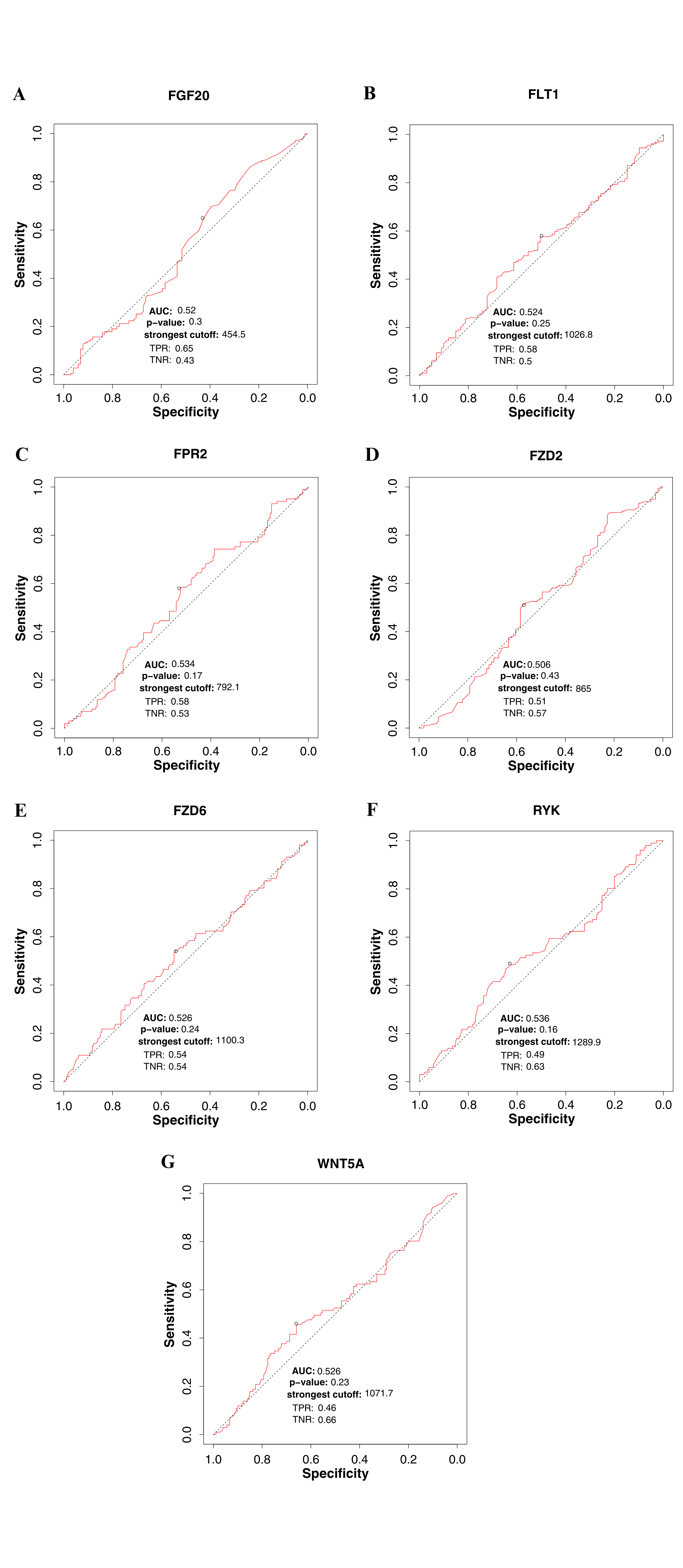

Supplement: Supplementary file 1 — Supplementary Material 1. [file 12964_2025_2424_MOESM1_ESM.zip › Supplementary/Sup 8.png]

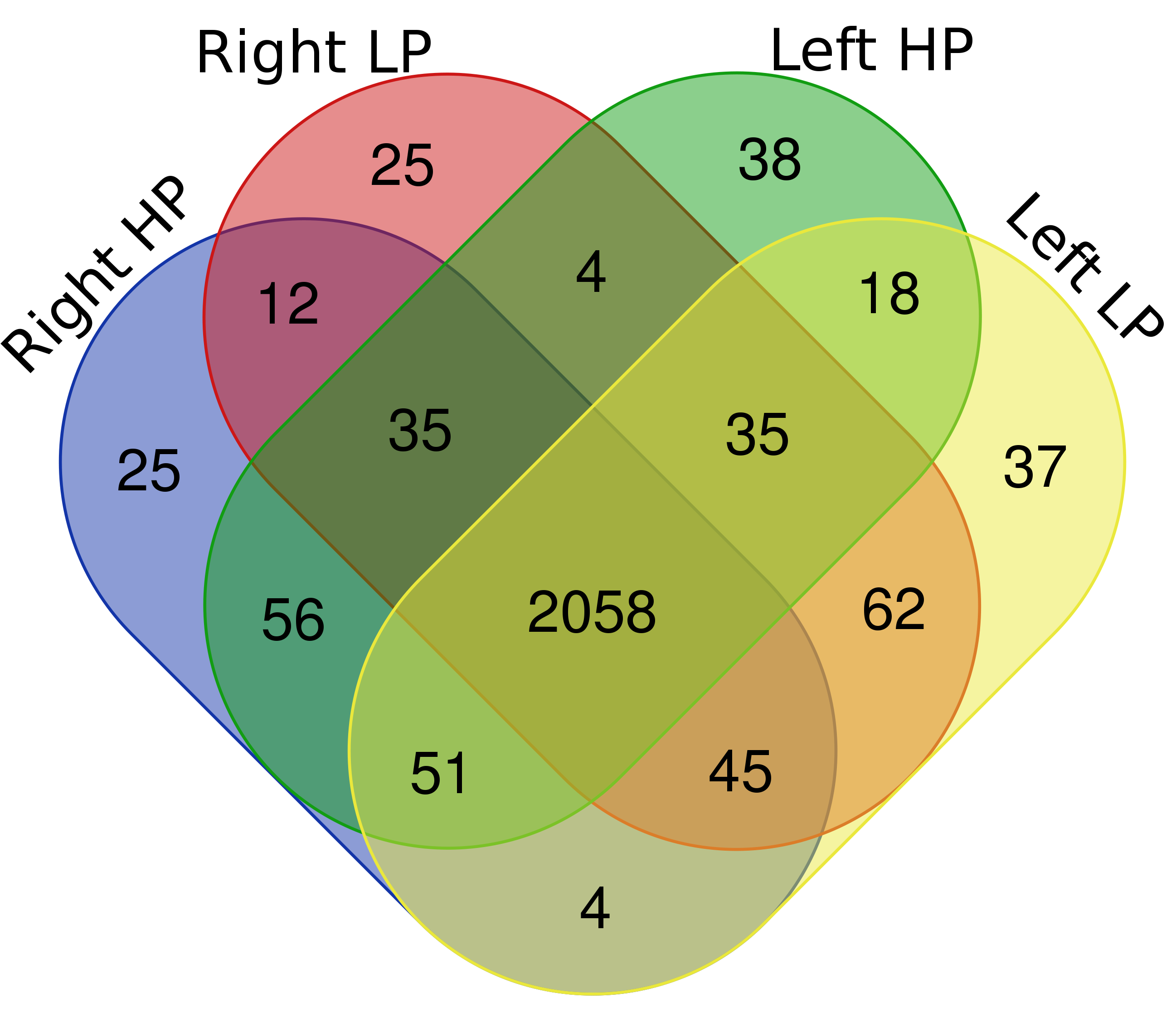

Supplement: Supplementary file 1 — Supplementary Material 1. [file 12964_2025_2424_MOESM1_ESM.zip › Supplementary/Sup 9.png]
